# Threshold dynamics of a reaction–advection–diffusion schistosomiasis epidemic model with seasonality and spatial heterogeneity

**DOI:** 10.1007/s00285-024-02097-6

**Published:** 2024-04-30

**Authors:** Peng Wu, Yurij Salmaniw, Xiunan Wang

**Affiliations:** 1 School of Sciences, Hangzhou Dianzi University, Hangzhou, 310018 China; 2 Department of Mathematical and Statistical Sciences, University of Alberta, Edmonton, T6G 2G1 Canada; 3 Present Address: Mathematical Institute, University of Oxford, Woodstock Road, Oxford, OX2 6GG UK; 4 Department of Mathematics, University of Tennessee at Chattanooga, Chattanooga, TN 37403 USA

**Keywords:** Schistosomiasis, Seasonality, Spatial heterogeneity, Reaction–advection–diffusion equations, Basic reproduction number, Threshold dynamics, 92D30, 45J50

## Abstract

Most water-borne disease models ignore the advection of water flows in order to simplify the mathematical analysis and numerical computation. However, advection can play an important role in determining the disease transmission dynamics. In this paper, we investigate the long-term dynamics of a periodic reaction–advection–diffusion schistosomiasis model and explore the joint impact of advection, seasonality and spatial heterogeneity on the transmission of the disease. We derive the basic reproduction number ${\mathcal {R}}_0$ and show that the disease-free periodic solution is globally attractive when ${\mathcal {R}}_0<1$ whereas there is a positive endemic periodic solution and the system is uniformly persistent in a special case when ${\mathcal {R}}_0>1$. Moreover, we find that ${\mathcal {R}}_0$ is a decreasing function of the advection coefficients which offers insights into why schistosomiasis is more serious in regions with slow water flows.

## Introduction

### Impact of schistosomiasis: worldwide and in China

Schistosomiasis, also referred to as bilharzia, is a parasitic disease which threatens public health and brings significant economic concerns, infecting approximately 200 million people worldwide. According to the World Health Organization (WHO), it has been reported in 78 countries and is endemic in 51 countries (World Health Organization 2022).

Schistosomiasis is an acute and chronic disease caused by a particular genus of blood flukes, referred to as *Schistosoma*. There are two main types of schistosomiasis: intestinal schistosomiasis, which is mainly caused by *Schistosoma mansoni* (Africa and other countries) or *Schistosoma japonicum* (China, Indonesia, the Philippines); and urinary schistosomiasis, caused by *Schistosoma haematobium* (Africa, the Middle East). Many people with schistosomiasis will not have any symptoms, or will not experience any symptoms sometimes for many months. For others, acute symptoms include fever, rash, a cough, diarrhoea, muscle and joint pain, or abdominal pain. When left untreated, eggs may travel to different parts of the body causing more serious issues. This is referred to as chronic schistosomiasis. Symptoms include anaemia, cystitis, persistent coughing, wheezing and shortness of breath, and in some cases, seizures and even paralysis. When left untreated, permanent organ damage is possible (World Health Organization 2022; National Health Service 2021). In children, who comprise approximately 123 million of all cases, the disease causes deficient growth and cognitive development (Osakunor et al. 2018). For a more complete exploration of the impacts of Schistosomiasis in humans, we refer readers to Colley et al. (2014), Rinaldo et al. (2021)

While direct economic impacts of schistosomiasis is difficult to quantify, a recent study estimates that the elimination of the disease in Burkina Faso would increase average crop yields by 7–32% (Rinaldo et al. 2021). Moreover, it is noted that “villages in proximity of large-scale dams suffer an average [agricultural] yield loss of around 20%”. It is therefore important to understand how proximity to water sources effects the spread of the disease.

Despite a majority of cases occurring in South Africa (World Health Organization 2022), the disease also remains endemic in some provinces of China, including Hubei, Hunan, Jiangxi, and Anhui; moreover, the monthly data of schistosomiasis cases in Hubei, Hunan and Anhui recorded by Chinese Center for Disease Control and Prevention (China CDC) display a seasonal pattern (Li et al. 2017). Of note, the Hubei province is located in the middle of the Yangtze River where the parasite is known to occupy. Global warming has also been considered as an exacerbating factor in the prevalence and spread of the disease (De Leo et al. 2020).

### Mechanism of schistosomiasis transmission

Individuals become infected through direct contact with contaminated water. Transmission can also occur when people already suffering from the disease contaminate existing freshwater sources via their excrement, in which the eggs can be found. The eggs then hatch and may come in contact with the skin of other individuals using common water sources. This means that it is not possible to catch the infection directly from someone else who already has the disease. Interestingly, the transmission of schistosomiasis involves the action of an intermediate host - in this case, *Oncomelania hupensis*, a freshwater snail (Colley et al. 2014). Adult parasites do not only infect humans; they may also reside within mammals such as cattle, water buffalo, and many rodent species (De Leo et al. 2020).

Contamination occurs when the urine or fecal matter of infected individuals enters the water source. The eggs, contained within the excrement, then hatch as *miracidia* and are able to occupy the freshwater snail and develop and multiply within its intermediate host. The parasite is then able to leave the snail as *cercariae* and enter the fresh water source where it can survive for a number of days (Colley et al. 2014). When susceptible humans (or other mammals) wade, swim, bathe, or even perform domestic chores within the water, the cercariae are able to penetrate the skin and infect the host (World Health Organization 2022). In the preceding weeks, the parasites mature into adult worms, living in the blood vessels of the body. Here, the worms are then able to produce eggs. These eggs make their way to the bladder and/or intestine, at which point they are deposited through excrement, and the cycle may continue. Transmission can also occur if unfiltered water is taken from a contaminated body of water to be used for showers or hand washing. When left untreated, the worms are able to continue laying eggs for up to several years, or in rare cases, upwards of 40 years (Colley et al. 2014).

### Prevention and control measures

Despite the debilitating impact of schistosomiasis on both individuals and communities, effective treatment and prevention measures exist and are quite well-known. The most basic means of prevention is to avoid contact with contaminated water, though this is not often an adequate solution when alternative water sources are unavailable. While no vaccine exists (Tebeje et al. 2016), other prevention measures include improving access to safe water, improved sanitation measures, educating the public on effective hygienic practices, as well as direct control of existing snail populations. For individuals already infected, *Praziquantel* tablets are an effective form of treatment to all species of Schitosoma (Zwang and Olliaro 2017; Centers for Disease Control and Prevention 2020), though the timing of application is important since it is most effective against adult worms (Centers for Disease Control and Prevention 2020). Some argue that new or additional drugs should be developed and utilized to best treat at risk populations (Bergquist et al. 2017). Unfortunately, an approved formulation for preschool aged children (1–4 years old) does not currently exist (World Health Organization 2022; Zwang and Olliaro 2017) and is noted as a practical limitation in the routine treatment of young children (Zwang and Olliaro 2017).

The primary method of prevention and reduction is the mass administration of drugs like Praziquantel. In high risk communities ($\ge$ 50% prevalence in school-aged children), it is recommended to treat all school-aged children once per year along with treating adults considered at risk; for moderate risk communities (10–50% prevalence in school-aged children), treat all school-aged children once every two years along with treatment for adults in special groups only; for low risk communities (<10% prevalence), treat all school-aged children twice during their primary schooling age (Inobaya et al. 2014). This intervention is relatively low cost and yields substantial positive impact on affected communities (Inobaya et al. 2014). However, despite the low cost, the magnitude of infected individuals can make this strategy difficult to sustain. In fact, prevalence may reach equivalent levels as before the intervention within 18–24 months after ceasing treatment (Gray et al. 2010). Moreover, issues with compliance in some countries has been known to reduce the benefits of this strategy (Tallo et al. 2008). Drug tolerance has also been raised as a concern when the drug is administered repeatedly (Humphries et al. 2012).

Due to these difficulties, it is important to implement multiple measures to control schistosomiasis prevalence. One such alternative measure is to control the population of the intermediate host. A chemical compound can be used to reduce the snail population, commonly referred to as *molluscicides*, with varying composition and efficacy (Inobaya et al. 2014). In order for these efforts to be effective, repeated treatments are necessary, making it a time-consuming and expensive alternative, not to mention possible unintentional effects on other local species.

For other approaches, such as improved health education and behavioural changes, reasonable alternatives must be provided in order for the change to occur. For example, a water recreation area was built in Ghana to discourage youth from going into the local river, resulting in significant decrease in new cases of Schistosomiasis (Kosinski et al. 2012). Another example includes providing appropriate vessels to contain excrement from fishing boats in China in order to prevent depositing directly into the water (Wang et al. 2009). Together, these efforts result in a decrease in mortality, prevalence, and severity of infection (Inobaya et al. 2014).

### Existing modelling efforts

Since the earliest schistosomiasis modelling works by Macdonald (1965) and Hairston (1965), numerous mathematical frameworks have been proposed to investigate the transmission dynamics of schistosomiasis (see, e.g., Woolhouse (1991, 1992) and the references therein for a systematic review of modelling efforts up to the early 1990’s; see, e.g., Anderson et al. (2016) and the references therein for a more recent historical review of some existing modelling efforts). We discuss in more detail some notable efforts related to the work carried out in the current paper.

In 2002, Feng et al. (2002) formulated a schistosomiasis model with density dependence and infection age in snail population, and followed up with an estimation of parameters in 2004 (Feng et al. 2004). In 2011, Gao et al. (2011) studied a control problem for schistosomiasis transmission, considering in detail the influence of various control mechanisms on the stability of the endemic equilibrium. In 2016, to capture the effect of delays due to incubation or maturation times, Ding et al. (2016) consider a 6-dimensional system with five time delays and study the stability of the disease-free equilibrium; Ding, Liu et al. follow up with a study of the endemic equilibrium in 2019 (Ding et al. 2019). In order to incorporate the influence of seasonality on disease transmission, Li et al. (2017) proposed a periodic model of schistosomiasis in 2017, analysing the model dynamics and performing a sensitivity analysis of the basic reproduction number with respect to some key parameters. More recently, in 2020 Zhang and Zhao (2020) developed a periodic model for schistosomiasis with time-dependent delays to study the transmission dynamics of schistosomiasis with a case study in Hubei, China.

In the models discussed above, many biological and ecological mechanisms are considered. However, the transmission mechanisms of the disease usually involve economic, psychological and social factors in addition to the intrinsic disease biology and ecology. For example, more than 82% of infected individuals lived in lake and marshland regions along the Yangtze River (Li et al. 2017). Hence, to study the impact of spatial heterogeneity and seasonality on the disease transmission, a periodic reaction–diffusion model including seasonal and spatial dynamical behaviors in the process of the disease was recently developed in Wang et al. (2018), Wang and Zhao (2008), Liang et al. (2019). As noted previously, schistosomiasis results from contact with cercariae in water, and so the impact of water flow on the survival of miracidia (cercariae) and their parasitic ability cannot be ignored. Indeed, stationary water sources contain the highest cercarial density, though the parasite may be limited by its poor ability to move horizontally (Walz et al. 2015; Upatham 1973). On the other hand, flowing water can facilitate the long distance transport of the parasite (Walz et al. 2015). In some cases, it is found that slow moving water ($\sim$0.1 m/s) is beneficial to the spread of the parasite (Walz et al. 2015), though faster speeds (>0.13 m/s) may instead limit the ability of miracidia to infect the host snails (Upatham 1973). Therefore, advection of mircaida and cercariae as a key factor in schistosomiasis modeling should not be neglected. In fact, the relevant research works on reaction–advection–diffusion model have begun to study the impact of advection on disease transmission (Gu et al. 2014, 2015a, b; Tian and Ruan 2018; Cheng and Zheng 2021). In 2018, Tian and Ruan (2018) studied the traveling wave of an advection–reaction–diffusion competition system with double free boundaries modeling invasion and competition of $Aedes\ Albopictus$ and $Aedes\ Aegypti$ mosquitoes. In 2021, Cheng and Zheng (2021) investigated the dynamics and spreading speed of a reaction–diffusion system with advection modeling West Nile virus.

Inspired by the above works, in this paper we consider the effect of advective forces in the periodic schistosomiasis model (Li et al. 2017), in addition to the effect of the diffusion of individuals (humans, snails, miracidia and cercariae) in heterogeneous environments. Consideration of heterogeneous environments has been applied in a wide variety of other reaction–diffusion settings, and can allow modellers to include differences in human activity or climatic factors such as local rain fall. Non-constant rates of diffusion extend further the generality and, ideally, the applicability of models to account for differences in movement behaviour, such as the influence of the presence of other organisms affecting the movement of the parasite. To the best of our knowledge, the threshold dynamics of the reaction–advection–diffusion schistosomiasis model has rarely been studied. Our goal is to gain a deeper understanding of the influence of heterogeneous environments, seasonal infection, and rates of advection on schistosomiasis transmission.

The remainder of this paper is organized as follows. We formulate our reaction–advection–diffusion schistosomiasis model with seasonality and spatial heterogeneity in Sect. 2. In Sect. 3, we present the preliminaries including local and global existence of the solution and the ultimate boundedness of the system. In Sect. 4, we derive the basic reproduction number ${\mathcal {R}}_0$ of the system by applying the next generation operator. We investigate the threshold dynamics in terms of ${\mathcal {R}}_0$ in Sect. 5. In Sect. 6, we carry out numerical simulations to explore the influence of advection, seasonality and spatial heterogeneity on disease transmission dynamics, complimenting the theoretical analysis. Finally, we give a brief discussion in Sect. 7.

## Model formulation

To study the joint impact of seasonality and the diffusion and advection of miracidia and cercariae in rivers on the transmission of schistosomiasis, we extend the model given in Li et al. (2017) as follows:

2.1 $$\begin{aligned} {\left\{ \begin{array}{ll} \frac{\partial S_1}{\partial t}=\Lambda _1-\alpha _1(t,y)S_1\frac{C}{C+B}-\delta _1S_1+\frac{\partial }{\partial y}\left( d_1(y)\frac{\partial S_1}{\partial y}\right) ,\\ \frac{\partial I_1}{\partial t}=\alpha _1(t,y)S_1\frac{C}{C+B}-(\delta _1+\sigma )I_1+\frac{\partial }{\partial y}\left( d_2(y)\frac{\partial I_1}{\partial y}\right) ,\\ \frac{\partial R}{\partial t}=\sigma I_1-\delta _1R+\frac{\partial }{\partial y}\left( d_3(y)\frac{\partial R}{\partial y}\right) ,\\ \frac{\partial S_2}{\partial t}=\Lambda _2(t)-\alpha _2(t,y)S_2\frac{M}{M+K}-\delta (t)S_2+\frac{\partial }{\partial y}\left( d_4(y)\frac{\partial S_2}{\partial y}\right) ,\\ \frac{\partial I_2}{\partial t}=\alpha _2(t,y)S_2\frac{M}{M+K}-\delta _2(t)I_2+\frac{\partial }{\partial y}\left( d_5(y)\frac{\partial I_2}{\partial y}\right) ,\\ \frac{\partial M}{\partial t}=\epsilon I_1+h_M(t,y)M\left( 1-\frac{M}{K_M}\right) -\delta _MM-\gamma _M\frac{\partial M}{\partial y}+\frac{\partial }{\partial y}\left( d_6(y)\frac{\partial M}{\partial y}\right) ,\\ \frac{\partial C}{\partial t}=\zeta I_2+h_C(t,y)C\left( 1-\frac{C}{B_C}\right) -\delta _CC-\gamma _C\frac{\partial C}{\partial y}+\frac{\partial }{\partial y}\left( d_7(y)\frac{\partial C}{\partial y}\right) , \end{array}\right. } \end{aligned}$$

for $(t,y) \in (0,\infty ) \times (0,H)$ and the initial condition

$$\begin{aligned} u(0,y)=u_0(y),\quad y\in [0,H],\quad \text {where}\ u=S_i,I_i,R,M,C,\ i=1,2. \end{aligned}$$

The growth dynamics are described by the forms introduced in Li et al. (2017). For the spatial components, we utilize Fick’s law of diffusion for each component. For susceptible and infected humans and snails, and for recovered humans, we assume the flux *J* for each population is proportional to the gradient of each population:

$$\begin{aligned} J = -d (y) \frac{\partial U}{\partial y}, \end{aligned}$$

where $d>0$ is a (possibly spatially dependent) diffusion rate corresponding to the concentration gradient of the population *U*. On the other hand, for the parasite populations, we assume that in addition to a diffusive flux, there is a drift downstream due to water flow proportional to the population density at some rate $\gamma \ge 0$:

$$\begin{aligned} J = -d(y) \frac{\partial U}{\partial y} + \gamma U \end{aligned}$$

For the boundary conditions, we assume that the human and snail populations satisfy a no-flux boundary condition at both ends of the river. In this case, this is equivalent to a homogeneous Neumann boundary condition at both ends of the river:

2.2 $$\begin{aligned} \begin{aligned}&\frac{\partial S_i}{\partial y}(t,z)=\frac{\partial I_i}{\partial y}(t,z)=\frac{\partial R}{\partial y}(t,z)=0,\quad z = 0,H,\ \ i=1,2,\ \ t>0. \end{aligned} \end{aligned}$$

On the other hand, the parasite populations are assumed to satisfy a no-flux boundary condition at the upstream end of the river, while satisfying a more general boundary condition at the downstream end of the river:

2.3 $$\begin{aligned} \begin{aligned}&d_6(0)\frac{\partial M}{\partial y}(t,0)-\gamma _M M(t,0)=d_7(0)\frac{\partial C}{\partial y}(t,0)-\gamma _C C(t,0)=0,\\&d_6(H)\frac{\partial M}{\partial y}(t,H)-\gamma _M M(t,H)=-b_M\gamma _M M(t,H),\\&d_7(H)\frac{\partial C}{\partial y}(t,H)-\gamma _C C(t,H)=-b_C\gamma _C C(t,H), \quad t>0. \end{aligned} \end{aligned}$$

**Fig. 1:**
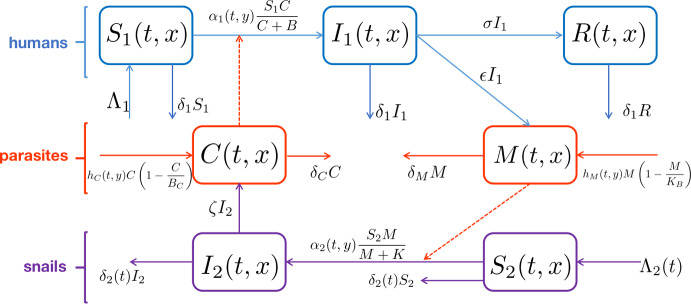
The schematic diagram of model (2.1)

This is reasonable as the parasite populations are incapable of swimming upstream through motility alone when one considers the size of the river in relation to the size of the parasites. Then, we can describe different scenarios at the mouth of the river depending on the particular qualities of the river considered. The parameters $b_M$ and $b_C$ respectively denote the relative loss rates of miracidia and cercariae due to water flux at the river’s downstream end. In particular, $b_M=0$ and $b_C=0$ represent the no-flux boundary condition; $b_M=1$ and $b_C=1$ yield the homogeneous Neumann boundary condition; and $b_M\rightarrow \infty$ and $b_C\rightarrow \infty$ lead to the homogeneous Dirichlet boundary condition (i.e., $M(t,H)=C(t,H)=0$). The case of $b_M\rightarrow \infty$ and $b_C\rightarrow \infty$ capture the situation where the river stream runs into the ocean so that the downstream area environment is hostile for the survival of miracidia and cercariae. These differing scenarios will depend on the particular nature of the downstream end of the river considered; a no-flux boundary condition may only be appropriate if the downstream end of the river has a still pool resulting in a negligible loss of parasite. In reality, it is more likely that $b_M$, $b_C$ are strictly positive. A schematic diagram of model (2.1) is shown in Fig. 1.

We now describe the components appearing in model (2.1), with a reference guide found in Table 2.1. The recruitment rate of susceptible human population is $\Lambda _1$ and we assume that there is no immigration for infected or recovered populations. In order to incorporate the influence of seasonal variations of human activities and parasitic worm population in water as well as the spatial heterogeneity of disease transmission, we assume that the transmission rate from cercariae to humans and that from miracidia to snails are time-periodic and space-dependent functions and denote them as $\alpha _1(t,y)$ and $\alpha _2(t,y)$, respectively. The densities of newly infected human individuals and snails per unit time at time *t* and location *y* are given by $\alpha _1(t,y)S_1\frac{C}{C+B}$ and $\alpha _2(t,y)S_2\frac{M}{M+K}$, respectively, where *B* and *K* are the corresponding half saturation constants. The natural death rate and recovery rate for humans is $\delta _1$ and $\sigma$, respectively. Since the snail population also varies seasonally in water, we assume that both the recruitment rate ($\Lambda _2(t)$) and the natural death rate ($\delta (t)$, $\delta _2(t)$) of snails are continuous and time-periodic functions. Note that the death rate of infected snails $\delta _2(t)$ may be different than that of susceptible snails $\delta (t)$, which we include as a possibility here. The population of miracidia increases when the eggs shed from infected people hatch in the water and the shedding rate is denoted by $\epsilon$. The population of cercariae increases when they are released from snails into water and the releasing rate is denoted by $\zeta$. In addition to the shedding from infected people and releasing from snails, we also assume logistic growth for miracidia and cercariae in water. The intrinsic growth rates of miracidia and cercariae are given by $h_M(t,y)$ and $h_C(t,y)$, respectively. The maximum capacities for miracidia and cercariae are denoted by $K_M$ and $B_C$, respectively. The natural death rates of miracidia and cercariae are $\delta _M$ and $\delta _C$, respectively. In principle, $\delta _M$ and $\delta _C$ could also be periodic functions of time; however, the survival time of both miracidia and cercariae are known to be significantly smaller (hours) than that of snails or humans (months or years) (Souza 2022). We therefore assume that these rates are constant in time.

The diffusion of each population is described by the term $\frac{\partial }{\partial y}(d_j(y)\frac{\partial u}{\partial y})$, $j=1$, 2,..., 7, respectively, where $d_j(y)$ is the location dependent diffusion rate. As noted above, our diffusive term is of Fickian form, which corresponds to neutral transition probabilities (as opposed to attractive or repulsive probabilities) (Potapov et al. 2014); other forms of diffusion may also be appropriate, but we do not explore these alternatives any further here.

Since schistosomiasis is caused by miracidia and cercariae which are swiming in rivers, the impact of water flow on their survival and movement cannot be ignored. Similar to Wang et al. (2022), we introduce the advection terms $-\gamma _M\frac{\partial M}{\partial y}$ and $-\gamma _C\frac{\partial C}{\partial y}$ for miracidia and cercariae, respectively, where $\gamma _M>0$ and $\gamma _C>0$ are the advection rates. Note that positive advection rates correspond to a constant flow from upstream to downstream.

**Table 1:** The biological interpretations of the parameters of system (2.1)

Parameters	Description
$\Lambda _1$	Recruitment of susceptible human individuals
$\alpha _1(t,y)$	Transmission rate fromfrom cercariae to humans
$\alpha _2(t,y)$	Transmission rate from miracidia to snails
$\delta _1$	Natural death rate of humans
$\delta (t)$	Death rate of susceptible snails
$\delta _2(t)$	Death rate of infected snails
$d_j(x)$	Diffusion rate of population in the *j*-th compartment, $j=1, 2,\ldots , 7$
$\delta _M$	Natural death rate of miracidia
$\delta _C$	Natural death rate of cercariae
$\sigma$	Recovery rate of infected human individuals
$\epsilon$	Shedding rate of parasitic eggs from humans into water
$\zeta$	Releasing rate of cercariae from snails into water
$\gamma _M$	Advection coefficient of miracidia
$\gamma _C$	Advection coefficient of cercariae
$b_M$	Relative rate of miracidia loss at the river’s downstream end
$b_C$	Relative rate of cercariae loss at the river’s downstream end
*K*(*B*)	Half saturation constant of infection by miracidia (cercariae)
$K_M (B_C)$	Maximal capacity of miracidia (cercariae) in the river
$h_M(t,y) (h_C(t,y))$	Intrinsic growth rate of miracidia (cercariae)

## Preliminaries

From the perspective of biological application, we make the following reasonable assumptions:

1. All model parameters are positive;
2. $d_j(y)\ (j=1, 2,\ldots , 7)$ are positive ${\textbf {C}}^1$-functions with respect to $y\in [0,H]$;
3. $$u\in {\textbf {C}}({\mathbb {R}}_+,[0,H])$$, $$u=S_i,I_i,R,M,C$$, $$i=1,2$$;
4. The initial values $(M_0,C_0)\not \equiv 0$ on [0, *H*];
5. $h_M(t,y)$, $h_C(t,y)$ and $\alpha _i(t,y)$, $i=1, 2$, are nontrivial, nonnegative, Hölder continuous functions on ${\mathbb {R}}\times [0,H]$ and $\omega$-periodic with respect to *t*;
6. $\Lambda _2(t)$ and $\delta _2(t)$ are nonnegative and $\omega$-periodic with respect to *t*.

For convenience, we denote by

$$\begin{aligned}{} & {} w_1(t,y):=S_1(t,y),\quad w_2(t,y):=I_1(t,y),\quad w_3(t,y):=R(t,y),\\{} & {} w_4(t,y):=S_2(t,y),\quad w_5(t,y):=I_2(t,y),\\{} & {} w_6(t,y):=M(t,y)\exp \left\{ -\int _0^y\frac{\gamma _M}{d_6(x)}dx\right\} ,\\{} & {} w_7(t,y):=C(t,y)\exp \left\{ -\int _0^y\frac{\gamma _C}{d_7(x)}dx\right\} , \end{aligned}$$

in which case system (2.1) can be rewritten as follows:

3.1 $$\begin{aligned} {\left\{ \begin{array}{ll} \frac{\partial w_1}{\partial t}=\Lambda _1-\alpha _1(t,y)\frac{w_1w_7}{w_7+\hat{B}(y)}-\delta _1w_1+\frac{\partial }{\partial y}\left( d_1(y)\frac{\partial w_1}{\partial y}\right) ,\\ \frac{\partial w_2}{\partial t}=\alpha _1(t,y)\frac{w_1w_7}{w_7+\hat{B}(y)}-(\delta _1+\sigma )w_2+\frac{\partial }{\partial y}\left( d_2(y)\frac{\partial w_2}{\partial y}\right) ,\\ \frac{\partial w_3}{\partial t}=\sigma w_2-\delta _1w_3+\frac{\partial }{\partial y}\left( d_3(y)\frac{\partial w_3}{\partial y}\right) ,\\ \frac{\partial w_4}{\partial t}=\Lambda _2(t)-\alpha _2(t,y)\frac{w_4w_6}{w_6+\hat{K}(y)}-\delta (t)w_4+\frac{\partial }{\partial y}\left( d_4(y)\frac{\partial w_4}{\partial y}\right) ,\\ \frac{\partial w_5}{\partial t}=\alpha _2(t,y)\frac{w_4w_6}{w_6+\hat{K}(y)}-\delta _2(t)w_5+\frac{\partial }{\partial y}\left( d_5(y)\frac{\partial w_5}{\partial y}\right) ,\\ \frac{\partial w_6}{\partial t}=\epsilon w_2\exp \left\{ -\int _0^y\frac{\gamma _M}{d_6(x)}dx\right\} + h_M(t,y)w_6\left( 1-\frac{w_6}{\hat{K}_M(y)}\right) -\delta _M(t)w_6\\ \quad \quad \quad +\gamma _M\frac{\partial w_6}{\partial y}+\frac{\partial }{\partial y}\left( d_6(y)\frac{\partial w_6}{\partial y}\right) ,\\ \frac{\partial w_7}{\partial t}=\zeta w_5\exp \left\{ -\int _0^y\frac{\gamma _C}{d_7(x)}dx\right\} +h_C(t,y)w_7 \left( 1-\frac{w_7}{\hat{B}_C(y)}\right) -\delta _C(t)w_7\\ \quad \quad \quad +\gamma _C\frac{\partial w_7}{\partial y}+\frac{\partial }{\partial y}\left( d_7(y)\frac{\partial w_7}{\partial y}\right) , \end{array}\right. } \end{aligned}$$

for $$(t,y) \in (0,\infty ) \times (0,H)$$, where

$$\begin{aligned}&\hat{B}(y):=B\exp \left\{ -\int _0^y\frac{\gamma _C}{d_7(x)}dx\right\} , \quad \hat{K}(y):=K\exp \left\{ -\int _0^y\frac{\gamma _M}{d_6(x)}dx\right\} , \\&\hat{B}_C(y):=B_C\exp \left\{ -\int _0^y\frac{\gamma _C}{d_7(x)}dx\right\} , \quad \hat{K}_M(y):=K_M\exp \left\{ -\int _0^y\frac{\gamma _M}{d_6(x)}dx\right\} . \end{aligned}$$

We then have the initial and boundary conditions

$$\begin{aligned} \begin{aligned}&w_j(0,y)=w_{j0}(y)\ge 0,\quad \quad \quad \quad j=1, 2,\ldots , 7,\ y\in [0,H],\\&\frac{\partial w_k}{\partial y}(t,0)=\frac{\partial w_k}{\partial y}(t,H)=0,\quad \quad k=1, 2,\ldots , 5,\\&\frac{\partial w_6}{\partial y}(t,0)= d_6(H)\frac{\partial w_6}{\partial y}(t,H)+b_M\gamma _Mw_6(t,H)=0,\\&\frac{\partial w_7}{\partial y}(t,0)= d_7(H)\frac{\partial w_7}{\partial y}(t,H)+b_C\gamma _Cw_7(t,H)=0, \end{aligned} \end{aligned}$$

for all $$t>0$$.

We first prove that the solution of system (3.1) exists locally and is positive in the positive cone of ${\textbf {A}}={\textbf {C}}({\mathbb {R}}^6,[0,H])$, i.e., ${\textbf {A}}^+={\textbf {C}}({\mathbb {R}}^6_+,[0,H])$. Let ${\textbf {X}}={\textbf {C}}({\mathbb {R}},[0,H])$ and ${\textbf {X}}^+={\textbf {C}}({\mathbb {R}}_+,[0,H])$. Define

$$\begin{aligned} {\mathscr {A}}(t,\tau ):=\text{ diag }\left\{ {\mathscr {A}}_1(t,\tau ),{\mathscr {A}}_2(t,\tau ), {\mathscr {A}}_3(t,\tau ),{\mathscr {A}}_4(t,\tau ),{\mathscr {A}}_5(t,\tau ),{\mathscr {A}}_6(t,\tau ), {\mathscr {A}}_7(t,\tau )\right\} , \end{aligned}$$

where ${\mathscr {A}}_j(t,\tau ):{\textbf {X}}^+\rightarrow {\textbf {X}}^+$, $j=1, 2,\ldots , 7$, are the operators associated with

$$\begin{aligned} \begin{aligned} \frac{\partial w_1}{\partial y}=&-\delta _1w_1+\frac{\partial }{\partial y}\left( d_1(y)\frac{\partial w_1}{\partial y}\right) ,\quad \frac{\partial w_2}{\partial y}=-\delta _1w_2+\frac{\partial }{\partial y}\left( d_2(y)\frac{\partial w_2}{\partial y}\right) ,\\ \frac{\partial w_3}{\partial y}=&-\delta _1w_3+\frac{\partial }{\partial y}\left( d_3(y)\frac{\partial w_3}{\partial y}\right) ,\\ \frac{\partial w_l}{\partial y}=&-\delta _2(t)w_l+\frac{\partial }{\partial y}\left( d_l(y)\frac{\partial w_l }{\partial y}\right) ,\ l=4,5,\\ \frac{\partial w_6}{\partial y}=&-\delta _Mw_6+\gamma _M\frac{\partial w_6}{\partial y}+\frac{\partial }{\partial y}\left( d_6(y)\frac{\partial w_6}{\partial y}\right) ,\\ \frac{\partial w_7}{\partial y}=&-\delta _Cw_7+\gamma _C\frac{\partial w_7}{\partial y}+\frac{\partial }{\partial y}\left( d_7(y)\frac{\partial w_7}{\partial y}\right) , \end{aligned} \end{aligned}$$

and

$$\begin{aligned}{} & {} \frac{\partial w_k}{\partial y}(t,0)=\frac{\partial w_k}{\partial y}(t,H)=0,\quad k=1, 2,\ldots , 5,\\{} & {} \frac{\partial w_6}{\partial y}(t,0)= d_6(H)\frac{\partial w_6}{\partial y}(t,H)+b_M\gamma _Mw_6(t,H)=0,\\{} & {} \frac{\partial w_7}{\partial y}(t,0)= d_7(H)\frac{\partial w_7}{\partial y}(t,H)+b_C\gamma _Cw_7(t,H)=0. \end{aligned}$$

From Wang et al. (2022) (see also, e.g., Martin and Smith (1990)), we know that ${\mathscr {A}}_j(t,\tau )$ is a strongly positive and compact operator on ${\textbf {X}}$ for $(t,\tau )\in {\mathbb {R}}^2$ and $t>\tau$. Denote

$$\begin{aligned} {\mathscr {B}}:=({\mathscr {B}}_1,{\mathscr {B}}_2,{\mathscr {B}}_3,{\mathscr {B}}_4,{\mathscr {B}}_5, {\mathscr {B}}_6,{\mathscr {B}}_7):[0,\infty )\times {\textbf {A}}^+\rightarrow {\textbf {A}} \end{aligned}$$

by

$$\begin{aligned}{} & {} {\mathscr {B}}_1(t,\psi ):=\Lambda _1-\alpha _1(t,\cdot )\frac{\psi _1\psi _7}{\psi _7+\hat{B}(\cdot )},\, {\mathscr {B}}_2(t,\psi ):=\alpha _1(t,\cdot )\frac{\psi _1\psi _7}{\psi _7+\hat{B}(\cdot )},\\{} & {} {\mathscr {B}}_3(t,\psi ):=\sigma \psi _2,\,{\mathscr {B}}_4(t,\psi ):=\Lambda _2(t)-\alpha _2(t,\cdot )\frac{\psi _4\psi _6}{\psi _6+\hat{K}(\cdot )},\\{} & {} {\mathscr {B}}_5(t,\psi ):=\alpha _2(t,\cdot )\frac{\psi _4\psi _6}{\psi _6+\hat{K}(\cdot )},\\{} & {} {\mathscr {B}}_6(t,\psi ):=\epsilon \psi _2\exp \left\{ -\int _0^y\frac{\gamma _M}{d_6(x)}dx\right\} +h_M(t,\cdot )\left( 1-\frac{\psi _6}{\hat{K}_M(y)}\right) ,\\{} & {} {\mathscr {B}}_7(t,\psi ):=\zeta \psi _5\exp \left\{ -\int _0^y\frac{\gamma _C}{d_7(x)}dx\right\} +h_C(t,\cdot )\left( 1-\frac{\psi _7}{\hat{B}_C(y)}\right) , \end{aligned}$$

for $\psi =(\psi _1,\psi _2,\ldots ,\psi _7)\in {\textbf {A}}^+$ and $t\ge 0$. Then system (3.1) can be rewritten as follows:

3.2 $$\begin{aligned} \frac{\text{ d }w}{\text{ d }t}={\mathscr {T}}(t)w+{\mathscr {B}}(t,w),\ t>0, \ w_0=\psi , \end{aligned}$$

where ${\mathscr {T}}:=\text{ diag }\{{\mathscr {T}}_1,\ldots ,{\mathscr {T}}_7\}$ and ${\mathscr {T}}_j$ is given by

$$\begin{aligned} \begin{aligned} {\mathscr {T}}_1\psi =&-\delta _1\psi +\frac{\partial }{\partial y}\left( d_1(y)\frac{\partial \psi }{\partial y}\right) ,\ \forall \ \psi \in D({\mathscr {T}}_1),\psi \in {\textbf {C}}^2([0,H]):\frac{\partial \psi }{\partial y}=0,\\&y=0,H,\\ {\mathscr {T}}_2\psi =&-\delta _1\psi +\frac{\partial }{\partial y}\left( d_2(y)\frac{\partial \psi }{\partial y}\right) ,\ \forall \ \psi \in D({\mathscr {T}}_2),\psi \in {\textbf {C}}^2([0,H]):\frac{\partial \psi }{\partial y}=0,\\&y=0,H,\\ {\mathscr {T}}_3\psi =&-\delta _1\psi +\frac{\partial }{\partial y}\left( d_3(y)\frac{\partial \psi }{\partial y}\right) ,\ \forall \ \psi \in D({\mathscr {T}}_3),\psi \in {\textbf {C}}^2([0,H]):\frac{\partial \psi }{\partial y}=0,\\&y=0,H,\\ {\mathscr {T}}_4\psi =&-\delta (t)\psi +\frac{\partial }{\partial y}\left( d_4(y)\frac{\partial \psi }{\partial y}\right) ,\ \forall \ \psi \in D({\mathscr {T}}_4),\psi \in {\textbf {C}}^2([0,H]):\frac{\partial \psi }{\partial y}=0,\\&y=0,H,\\ {\mathscr {T}}_5\psi =&-\delta _2(t)\psi +\frac{\partial }{\partial y}\left( d_5(y)\frac{\partial \psi }{\partial y}\right) ,\ \forall \ \psi \in D({\mathscr {T}}_5),\psi \in {\textbf {C}}^2([0,H]):\frac{\partial \psi }{\partial y}=0,\\&y=0,H\\ {\mathscr {T}}_6\psi =&-\delta _M\psi +\gamma _M\frac{\partial \psi }{\partial y}+\frac{\partial }{\partial y}\left( d_6(y)\frac{\partial \psi }{\partial y}\right) ,\ \forall \ \psi \in D({\mathscr {T}}_6),\\&\psi \in {\textbf {C}}^2([0,H]):\frac{\partial \psi }{\partial y}(0)=0,\ d_{6}(H)\frac{\partial \psi }{\partial y}(H)+b_M\gamma _M\psi (H)=0,\\ {\mathscr {T}}_7\psi =&-\delta _C\psi +\gamma _C\frac{\partial \psi }{\partial y}+\frac{\partial }{\partial y}\left( d_7(y)\frac{\partial \psi }{\partial y}\right) ,\ \forall \ \psi \in D({\mathscr {T}}_7)\\&\psi \in {\textbf {C}}^2([0,H]):\frac{\partial \psi }{\partial y}(0)=0,\ d_{7}(H)\frac{\partial \psi }{\partial y}(H)+b_C\gamma _C\psi (H)=0, \end{aligned} \end{aligned}$$

where $D(\cdot )$ is a closed subset of $[0,\infty )\times {\textbf {X}}$. Thus, from Martin and Smith (1990, Corollary 4 and Theorem 1), we can verify that system (3.1) has a unique solution $w(t,\cdot ,\psi )$ for $t\in [0,t_\psi ]$, $t_\psi \le \infty$. Similar to Wang et al. (2022, Theorem 2.1), we have the following result.

### Theorem 3.1

System (3.1) admits a unique solution $w(t,\cdot ,\psi )$ for $t\in [0,\infty )$ with initial value $w_0=\psi$. Furthermore, the $\omega$-periodic semiflow ${\mathcal {P}}(t)$ on ${\textbf {A}}^+$ generated by system (3.1) is defined as ${\mathcal {P}}(t)\psi =w(t,\cdot ,\psi )$, and ${\mathcal {P}}(\omega )$ admits a strong global attractor in ${\textbf {A}}^+$, $t\ge 0$.

### Proof

We first set $t_\psi$ be the maximal existence time of the solution *w*. We follow the proof of Wang et al. (2022, Theorem 2.1). More precisely, by Wang et al. (2022, Lemma 6.1) if we can show that there exists a positive function $M_{\alpha }(\psi )$ such that

3.3 $$\begin{aligned} \vert \vert w(t,\cdot )\vert \vert _{L^{\alpha }(\Omega )}\le M_{\alpha }(\psi ),\ \forall t\in (0,t_\psi ), \end{aligned}$$

for some $\alpha$ satisfying certain conditions depending on the spatial dimension and the growth rates of the system, we immediately obtain that the solution exists for all time $t \in (0,\infty )$ and that there holds

$$\begin{aligned} \vert \vert w(t,\cdot )\vert \vert _{L^{\infty }(\Omega )}\le M_{\infty }(\psi ). \end{aligned}$$

In our case, we will choose $\alpha = 1$, which can readily be found to satisfy the necessary conditions given in the statement of Wang et al. (2022, Lemma 6.1). Our goal is to obtain

3.4 $$\begin{aligned} \int _{\Omega }w_j(t,y,\psi )dy\le N^*(\psi ),\ 1\le j\le 7, \end{aligned}$$

for some $$N^*(\psi )>0$$.

To this end, we let $\Omega =(0,H)$ and set $N_1(t,y,\psi )=\sum _{i=1}^3w_i(t,y,\psi )$. We immediately obtain the following inequality from system (3.1):

3.5 $$\begin{aligned} \frac{\text{ d }}{\text{ d }t}\int _{\Omega }N_1(t,y,\psi )dy=&\int _{\Omega }\Lambda _1dy -\delta _1N_1(t,y,\psi )dy-\sum _{i=1}^3\int _{\Omega }\frac{\partial }{\partial y}(d_i(y)\frac{\partial w_i}{\partial y})dy\nonumber \\ \le&\Lambda _1H-\int _{\Omega }\delta _1N_1(t,y,\psi )dy,\ t\in (0,t_\psi ). \end{aligned}$$

Applying Gronwall’s inequality we have

$$\begin{aligned} \int _{\Omega }N_1(t,y,\psi )dy\le e^{-\delta _it}\int _{\Omega }N_{1}(0,y,\psi )dy+\frac{\Lambda _1}{\delta _1}(1-e^{-\delta _1t}), \end{aligned}$$

where $N_1(0,\cdot ,\psi )=\sum _{i=1}^3\psi _i(\cdot )$, $\vert \Omega \vert =H$. Hence we have that

$$\begin{aligned} \int _{\Omega }N_1(t,y,\psi )dy\le M_1,\ t\in (0,t_\psi ), \end{aligned}$$

where $M_1:=M_1(\psi )>0$. Moreover, there holds

$$\begin{aligned} \limsup \limits _{t\rightarrow \infty }\int _{\Omega }N_1(t,y,\psi )dy\le \frac{\Lambda _1H}{\delta _1}. \end{aligned}$$

Next, we set $N_2(t,y,\psi )=w_4(t,y,\psi )+w_5(t,y,\psi )$ and obtain a similar bound for $N_2$. From system (3.1), we immediately obtain

3.6 $$\begin{aligned} \frac{\text{ d }}{\text{ d }t}\int _{\Omega }N_2(t,y,\psi )dy=&\int _{\Omega }\Lambda _2(t)dy {-}\delta _2(t)N_2(t,y,\psi )dy{-}\sum _{i=4}^5\int _{\Omega }\!\frac{\partial }{\partial y}(d_i(y)\frac{\partial w_i}{\partial y})dy \nonumber \\ \le&\Lambda _2(t)H-\int _{\Omega }\delta _2(t)N_2(t,y,\psi )dy,\ t\in (0,t_\psi ). \end{aligned}$$

We then consider the following linear periodic reaction–diffusion system

3.7 $$\begin{aligned} \left\{ \begin{aligned}&\frac{\partial S_2}{\partial t}=\Lambda _2(t)-\delta (t)S_2+\frac{\partial }{\partial y}\left( d_4(y)\frac{\partial S_2}{\partial y}\right) ,\ (t,y)\in (0,\infty )\times (0,H),\\&\frac{\partial S_2}{\partial y}(t,0)=\frac{\partial S_2}{\partial y}(t,H)=0, \hspace{2.5cm} t>0. \end{aligned} \right. \end{aligned}$$

From Zhang et al. (2015, Lemma 2.1), it is easy to obtain that system (3.7) has a unique $\omega$-periodic solution $\widehat{S}_2(t,\cdot )$ and it is globally attractive in ${\textbf {X}}^+$.

If we set $\int _{\Omega }N_2(t,y,\psi )dy={\textbf {N}}_2(t,\psi )$, from inequality (3.6) we have that $\frac{\text{ d }}{\text{ d }t}{} {\textbf {N}}_2(t,\psi )\le \Lambda _2(t)H-\delta _2(t){\textbf {N}}_2(t,\psi )$. By the comparison principle and Teng et al. (2008, Lemma 1), there holds

$$\begin{aligned} \int _\Omega N_2 (t,y,\psi ) \le M_2, \end{aligned}$$

where $M_2:= M_2 (\psi )>0$; moreover we obtain $\limsup _{t\rightarrow \infty }({\textbf {N}}_2(t)-\widehat{S}_2(t))\le 0$ which implies that

$$\begin{aligned} \limsup \limits _{t\rightarrow \infty }\int _{\Omega }N_2(t,y,\psi )dy\le \limsup \limits _{t\rightarrow \infty } \widehat{S}_2(t,\psi )\le \sup _{t\in [0,\omega ]}\widehat{S}_2(t,\psi ):=S^*_2(\psi ). \end{aligned}$$

Next, we obtain bounds for $w_6,w_7$. Note that

$$\begin{aligned} \begin{aligned}&\exp \left\{ -\int _0^y\frac{\gamma _M}{d_6(x)}dx\right\} \left( -\gamma _M\frac{\partial w_6}{\partial y}+\frac{\partial }{\partial y}\left( d_6(y)\frac{\partial w_6}{\partial y}\right) \right) \\&\quad =-\gamma _M\frac{\partial M}{\partial y}+\frac{\partial }{\partial y}\left( d_6(y)\frac{\partial M}{\partial y}\right) ,\\&\exp \left\{ -\int _0^y\frac{\gamma _C}{d_7(x)}dx\right\} \left( -\gamma _M\frac{\partial w_7}{\partial y}+\frac{\partial }{\partial y}\left( d_7(y)\frac{\partial w_7}{\partial y}\right) \right) \\&\quad =-\gamma _C\frac{\partial M}{\partial y}+\frac{\partial }{\partial y}\left( d_7(y)\frac{\partial C}{\partial y}\right) , \end{aligned} \end{aligned}$$

and so one has

$$\begin{aligned} \begin{aligned}&\frac{\text{ d }}{\text{ d }t}\int _{\Omega }\exp \left\{ -\int _0^y\frac{\gamma _M}{d_6(x)}dx\right\} w_6(t,y,\psi )dy\\&\quad \le \epsilon N_1+\frac{h_MK_MH}{4}-\delta _M\int _{\Omega }\exp \left\{ -\int _0^y\frac{\gamma _M}{d_6(x)}dx\right\} w_6(t,y,\psi )dy,\\&\frac{\text{ d }}{\text{ d }t}\int _{\Omega }\exp \left\{ -\int _0^y\frac{\gamma _C}{d_7(x)}dx\right\} w_7(t,y,\psi )dy\\&\quad \le \epsilon N_2+\frac{h_CB_CH}{4}-\delta _C\int _{\Omega }\exp \left\{ -\int _0^y\frac{\gamma _C}{d_7(x)}dx\right\} w_7(t,y,\psi )dy \end{aligned} \end{aligned}$$

for $t\in (0,t_\psi )$. Thus, applying Gronwall’s inequality once more, we obtain that there exists $M_6(\psi ),M_7(\psi )>0$ such that

$$\begin{aligned} \begin{aligned}&\int _{\Omega }w_6(t,y,\psi )dy\le \int _{\Omega }\exp \left\{ -\int _0^y\frac{\gamma _M}{d_6(x)} dx\right\} w_6(t,y,\psi )dy\le M_6(\psi ),\\&\int _{\Omega }w_7(t,y,\psi )dy\le \int _{\Omega }\exp \left\{ -\int _0^y\frac{\gamma _C}{d_7(x)} dx\right\} w_7(t,y,\psi )dy\le M_7(\psi ) \end{aligned} \end{aligned}$$

for $t\in (0,t_\psi )$. Therefore, there exists a $N^*(\psi )=\max \{M_1(\psi ),M_2(\psi ),M_6(\psi ),M_7(\psi )\}$ such that (3.4) holds. Similar to the proof in Wang et al. (2022, Theorem 2.1), we find that $w_i(t,\cdot )$, $1 \le i \le 7$, are uniformly bounded, i.e., $\vert \vert w_i (t,\cdot ,\psi ) \vert \vert _{L^\infty (\Omega )} \le M_\infty (\psi )$. Furthermore, we have shown that $w_j$ is ultimately bounded, i.e., $\limsup _{t\rightarrow \infty } w_i (t,y,\psi ) \le M^*$ for some $M^*>0$, uniformly for $y \in \Omega$.

Finally, we define a family of maps $\{{\mathcal {P}}(t)\}_{t\ge 0}:{\textbf {A}}^+\rightarrow {\textbf {A}}^+$ as ${\mathcal {P}}(t)\psi =w(t,\cdot ,\psi )$ for $\psi \in {\textbf {A}}^+$. It follows from Zhao (2017) that ${\mathcal {P}}(t):{\textbf {A}}^+\rightarrow {\textbf {A}}^+$ is an $\omega$-periodic semiflow. Based on the above discussions, we can verify that ${\mathcal {P}}(t)$ is ultimately bounded. Combining the compactness of ${\mathcal {P}}(t)$ with Zhao (2017, Theorem 1.1.3), we can verify that ${\mathcal {P}}(\omega )$ admits a strong global attractor in ${\textbf {A}}^+$. $\square$

## The basic reproduction number of system (3.1)

In this section, we are devoted to deriving the functional expression of the basic reproduction number of system (3.1). For this purpose, we first consider the following parabolic problem

4.1 $$\begin{aligned} {\left\{ \begin{array}{ll} \frac{\partial w_1}{\partial t}=\Lambda _1-\delta _1w_1+\frac{\partial }{\partial y}\left( d_1(y)\frac{\partial w_1}{\partial y}\right) ,\quad (t,y)\in (0,\infty )\times (0,H),\\ \frac{\partial w_1}{\partial y}(t,0)=\frac{\partial w_1}{\partial t}(t,H)=0, \hspace{2.5cm} t > 0. \end{array}\right. } \end{aligned}$$

It is obvious that system (4.1) has a globally attractive constant positive solution $S^*_1=\Lambda _1/\delta _1$; the disease-free periodic solution is then given by $E_0=(S^*_1,0,\widehat{S}_2(t),0,0,0)$, where $\widehat{S}_2(t)$ is the unique $\omega$-periodic globally attractive solution of system (3.7). We first set $(v_1,v_2,v_3,v_4)=(w_2,w_5,w_6,w_7)$. Then, the linearized system around $E_0$ is given by

4.2 $$\begin{aligned} {\left\{ \begin{array}{ll} \frac{\partial v_1}{\partial t}=S^*_1\alpha _1(t,y)\frac{v_4}{B}\exp \left\{ \int _0^y\frac{\gamma _C}{d_7(x)}dx\right\} -(\delta _1+\sigma )v_1+\frac{\partial }{\partial y}\left( d_2(y)\frac{\partial v_1}{\partial y}\right) ,\\ \frac{\partial v_2}{\partial t}=\widehat{S}_2(t)\alpha _2(t,y)\frac{v_3}{K}\exp \left\{ \int _0^y\frac{\gamma _M}{d_6(x)}dx\right\} -\delta (t)v_2+\frac{\partial }{\partial y}\left( d_5(y)\frac{\partial v_2}{\partial y}\right) ,\\ \frac{\partial v_3}{\partial t}=\epsilon v_1\exp \left\{ -\int _0^y\frac{\gamma _M}{d_6(x)}dx\right\} +\gamma _M\frac{\partial v_3}{\partial y}-\delta _Mv_3+\frac{\partial }{\partial y}\left( d_6(y)\frac{\partial v_3}{\partial y}\right) ,\\ \frac{\partial v_4}{\partial t}=\zeta v_2\exp \left\{ -\int _0^y\frac{\gamma _C}{d_7(x)}dx\right\} +\gamma _C\frac{\partial v_4}{\partial y}-\delta _Cv_4+\frac{\partial }{\partial y}\left( d_7(y)\frac{\partial v_4}{\partial y}\right) ,\\ \end{array}\right. } \end{aligned}$$

for $(t,y)\in [0,\infty )\times (0,H)$ with boundary condition

$$\begin{aligned}&\frac{\partial v_i}{\partial y}(t,0)=\frac{\partial v_i}{\partial y}(t,H)=\frac{\partial v_k}{\partial y}(t,0)=0,\quad i=1,2,\ k=3,4,\\&d_6(H)\frac{\partial v_3}{\partial y}(t,H)=-b_M\gamma _Mv_3(t,H),\\&d_7(H)\frac{\partial v_4}{\partial y}(t,H)=-b_C\gamma _Cv_4(t,H), \quad \quad \quad t > 0. \end{aligned}$$

We denote by ${\textbf {Y}}:={\textbf {C}}({\mathbb {R}}^4,[0,H])$ and ${\textbf {Y}}^+:={\textbf {C}}({\mathbb {R}}^4_+,[0,H])$ and define ${\textbf {C}}_{\omega }({\mathbb {R}},{\textbf {Y}})$ as the Banach space space consisting of all $\omega$-periodic and continuous functions equipped with the norm $\vert \vert \phi \vert \vert _{{\textbf {C}}_{\omega }({\mathbb {R}},{\textbf {y}})}:=\max _{\theta \in [0,\omega ]}\vert \vert \phi (\theta )\vert \vert _{{\textbf {Y}}}$, $\phi \in {\textbf {C}}_{\omega }({\mathbb {R}},{\textbf {Y}})$.

For $(t,y)\in {\mathbb {R}}\times [0,H]$, we set

$$\begin{aligned}&{\mathscr {F}}(t,y)\\&= \left[ \begin{array}{cccc} 0&{}0&{}0&{}S^*_1\alpha _1(t,y)/\hat{K}(y)\\ 0&{}0&{}\widehat{S}_2(t)\alpha _2(t,y)/\hat{B}(y)&{}0 \\ \epsilon \exp \left\{ -\int _0^y\frac{\gamma _M}{d_6(x)}dx\right\} &{}0&{}h_M(t,y)&{}0\\ 0&{}\zeta \exp \left\{ -\int _0^y\frac{\gamma _C}{d_7(x)}dx\right\} &{}0&{}h_C(t,y)\\ \end{array} \right] , \end{aligned}$$

and ${\mathscr {F}}(t):{\textbf {Y}}\rightarrow {\textbf {Y}}$ by $[{\mathscr {F}}(t)\phi ](y)={\mathscr {F}}(t,y)\phi (y)$ for $y\in [0,H], \phi \in {\textbf {Y}}$. Let

$$\begin{aligned} \begin{aligned} {\mathscr {V}}(t,y)&:=\text{ diag }\left\{ \frac{\partial }{\partial y}\left( d_2(y)\frac{\partial }{\partial y}\right) -(\delta _1+\sigma ),\ \frac{\partial }{\partial y}\left( d_5(y)\frac{\partial }{\partial y}\right) -\delta _2(t),\right. \\&\quad \left. \gamma _M\frac{\partial }{\partial y}+\frac{\partial }{\partial y}\left( d_6(y)\frac{\partial }{\partial y}\right) -\delta _M,\ \gamma _C\frac{\partial }{\partial y}+\frac{\partial }{\partial y}\left( d_7(y)\frac{\partial }{\partial y}\right) -\delta _C \right\} . \end{aligned} \end{aligned}$$

Then system (4.2) can be expressed as

4.3 $$\begin{aligned} \frac{\text{ d }v}{\text{ d }t}={\mathscr {F}}(t)v-{\mathscr {V}}(t)v. \end{aligned}$$

Obviously, for each ${\mathscr {F}}(t):{\textbf {Y}}\rightarrow {\textbf {Y}}$, we have ${\mathscr {F}}(t){\textbf {Y}}^+\subset {\textbf {Y}}^+$. Let $\Psi (t,\tau )=\text{ diag }\{{\mathscr {A}}_2(t,\tau )$, ${\mathscr {A}}_5(t,\tau )$, ${\mathscr {A}}_6(t,\tau )$, ${\mathscr {A}}_7(t,\tau )\}$, $t\ge \tau$ be the evolution operators with respect to to system $\text{ d }v/\text{d }t=-{\mathscr {V}}(t)v$. By the positivity of $\Psi (t,\tau )$, it follows that there exists two constants $\Gamma >0$ and $r>0$ such that $\vert \vert \Psi (t,\tau )\vert \vert \le \Gamma e^{-r(t-\tau )},t\ge \tau$. For $\tau \ge 0$, ${\mathscr {F}}(\tau )v(\tau )$ denotes the densities of newly infected individuals, infected snails, miracidia and cercariae at time $\tau$. Then $\Psi (t,\tau ){\mathscr {F}}(\tau )v(\tau )$ represents the total densities of those compartments who become infected at time $\tau$ and still survive at time *t*. Hence

$$\begin{aligned} \int _0^{\infty }\Psi (t,\tau ){\mathscr {F}}(\tau )v(\tau )d\tau =\int _0^{\infty } \Psi (t,t-\tau ){\mathscr {F}}(t-\tau )v(t-\tau )d\tau , \end{aligned}$$

is the density of accumulative infected individuals from time $\tau$ to time *t*.

If we denote the following linear operators on ${\textbf {C}}_{\omega }({\mathbb {R}},{\textbf {Y}})$ by

$$\begin{aligned} \begin{aligned}&[{\mathscr {L}}_1v](t):=\int _0^{\infty }\Psi (t,t-\tau ){\mathscr {F}}(t-\tau )v(t-\tau )d\tau ,\ t\in {\mathbb {R}}, v\in {\textbf {C}}_{\omega }({\mathbb {R}},{\textbf {Y}}),\\&[{\mathscr {L}}_2v](t):={\mathscr {F}}(t)\int _0^{\infty }\Psi (t,t-\tau )v(t-\tau )d\tau ,\ v\in {\textbf {C}}_{\omega }({\mathbb {R}},{\textbf {Y}}),\\&[{\mathscr {W}}_1v](t):=\int _0^{\infty }\Psi (t,t-\tau )v(t-\tau )d\tau , \ v\in {\textbf {C}}_{\omega }({\mathbb {R}},{\textbf {Y}}),\\&[{\mathscr {W}}_2v](t):={\mathscr {F}}(t)v(t), \ v\in {\textbf {C}}_{\omega }({\mathbb {R}},{\textbf {Y}}), \end{aligned} \end{aligned}$$

then we can verify that ${\mathscr {L}}_1={\mathscr {W}}_1\circ {\mathscr {W}}_2$ and ${\mathscr {L}}_2={\mathscr {W}}_2\circ {\mathscr {W}}_1$, which implies that ${\mathscr {L}}_1$ and ${\mathscr {L}}_2$ have the same spectral radius. From Zhao (2017, Chapter 3) and Bacaer and Guernaoui (2006), we can obtain that the basic reproduction number of system (3.1) is

$$\begin{aligned} {\mathcal {R}}_0=r({\mathscr {L}}_1)=r({\mathscr {L}}_2), \end{aligned}$$

where $r({\mathscr {L}}_1)$ and $r({\mathscr {L}}_2)$ denote the spectral radius of ${\mathscr {L}}_1$ and ${\mathscr {L}}_2$, respectively.

Set ${\mathcal {Q}}(t)$ be the solution map of system (4.3) on ${\textbf {Y}}$ and ${\mathcal {Q}}\phi =z(t,\cdot ,\phi )$, $v=(w_2,w_5,w_6,w_7)$. Then, ${\mathcal {Q}}(t)$ is the Poincaré map with respect to system (4.3). Denote by $r({\mathcal {Q}}(\omega ))$ the spectral radius of ${\mathcal {Q}}(\omega )$. Based on the discussions in Wang and Zhao (2008), we have the following Lemma.

### Lemma 4.1

For the basic reproduction number ${\mathcal {R}}_0$, one has

1. $r({\mathcal {Q}}(\omega ))<1$ if and only if  ${\mathcal {R}}_0<1$,
2. $r({\mathcal {Q}}(\omega ))=1$ if and only if  ${\mathcal {R}}_0=1$,
3. $r({\mathcal {Q}}(\omega ))>1$ if and only if  ${\mathcal {R}}_0>1$.

### Lemma 4.2

Set $\lambda =\frac{\ln r({\mathcal {Q}}(\omega ))}{\omega }$. Then, there exists a positive $\omega$-periodic function $v^*(t,y)$ such that $v^*(t,y)e^{\lambda t}$ is a solution of system (4.2).

According to Liang et al. (2019, Theorem 3.8), we have the following Lemma.

### Lemma 4.3

If ${\mathcal {R}}_0>0$, then the following eigenvalue problem

4.4 $$\begin{aligned} \begin{aligned}&\frac{\partial u}{\partial t}=-{\mathscr {V}}(t)u+\mu {\mathscr {F}}(t,y)u,\ (t,y)\in (0,\infty )\times (0,H),\\&u(0,y)=u(\omega ,y),\ x\in (0,H),\\&{\mathbb {B}}_bu=0,\ y=0,H,\ t>0, \end{aligned} \end{aligned}$$

has a spectral eigenvalue $\mu =1/{\mathcal {R}}_0$; that is, the unique solution of the spectral radius $r(W(\omega$,0, $\mu ))=1$ is $\mu =1/R_0$, where $\{W(t,\tau ,\mu ):t\ge \tau \}$ denotes the family of the operator $\Xi$ with respect to (4.4) with $u(0,y)=u(\omega ,y),\ x\in (0,H)$ and

$$\begin{aligned} {\mathbb {B}}_b=\textrm{diag}\left\{ \frac{\partial }{\partial y},\frac{\partial }{\partial y},d_6(y)\frac{\partial }{\partial y}+b_M\gamma _M\Xi _{\{y=H\}}(y),d_7(y)\frac{\partial }{\partial y}+b_C\gamma _C\Xi _{\{y=H\}}(y)\right\} \end{aligned}$$

where $\Xi (s)$ is the characteristics function.

In fact, an important research motivation of this paper is to introduce the advection term into the model to analyze the impact of water flow on disease transmission. Therefore, we give the following monotonicity proposition of basic reproduction number and the relative rates of miracidia and cercariae’s loss at lower reach end of river.

### Proposition 4.4

${\mathcal {R}}_0={\mathcal {R}}_0(b)$ is a strictly decreasing function with respect to $b\in [0,\infty )$, where $b=b_M,b_C$.

### Proof

The approach is similar to Wang et al. (2022, Proposition 3.5(ii)). We show the details for completeness. Let $\mu (b)$ be the principal eigenvalue of (4.4) with respect to $b\ge 0$. We consider the monotonicity of ${\mathcal {R}}_0(b)$ with respect to $b=b_M,b_C$. In fact, if $b_{1}>b_{2}$, then $\mu (b_1)>\mu (b_2)$. If it is not true, then $\mu (b_1)\le \mu (b_2)$ for some $b_1>b_2$. Let $u^{(i)}(t,y)$ be the positive eigenfunction of (4.4) with eigenvalue $\mu (b_i)$ for $i=1,2$. By the non-negativity of ${\mathscr {F}}$, and the fact that

$$\begin{aligned} d_6(H)\frac{\partial u^{(1)}}{\partial y}(t,H)+b_2\gamma _Mu^{(1)}(t,H)=(b_2-b_1)\gamma _Mu^{(1)}(t,H)<0, \end{aligned}$$

and

$$\begin{aligned} d_7(H)\frac{\partial u^{(1)}}{\partial y}(t,H)+b_2\gamma _Cu^{(1)}(t,H)=(b_2-b_1)\gamma _Cu^{(1)}(t,H)<0, \end{aligned}$$

it follows that $u^{(1)}$ satisfies

4.5 $$\begin{aligned} {\left\{ \begin{array}{ll} \frac{\partial u^{(1)}}{\partial t}\le -{\mathscr {V}}(t)u^{(1)}+\mu {\mathscr {F}}(t,y)u^{(1)},\quad (t,y)\in (0,\infty )\times (0,H),\\ u^{(1)}(0,y)=u^{(1)}(\omega ,y),\hspace{2.2cm} y\in (0,H),\\ {\mathbb {B}}_{b_2}u^{(1)}\le 0,\hspace{3.5cm} y=0,H,\ t>0. \end{array}\right. } \end{aligned}$$

Let ${\mathcal {Q}}^{(i)}(t)$ be the periodic semiflow of (4.4) with $\mu =\mu (b_i)$ and $b=b_i$, $i=1,2$. It is obvious that if $r({\mathcal {Q}}^{(i)}(\omega )=1$, from (4.5) we then have $u^{(1)}(t,y)\le [{\mathcal {Q}}^{(2)}(t)u^{(1)}(0,\cdot )](y),t\ge 0$, $y\in (0,H)$. Thus

4.6 $$\begin{aligned} u^{(1)}(0,\cdot )=u^{(1)}(\omega ,\cdot )\le {\mathcal {Q}}^{(2)}(\omega )u^{(1)}(0,\cdot ). \end{aligned}$$

In fact, we can verify that $u^{(1)}(0,\cdot )\ne {\mathcal {Q}}^{(2)}(\omega )u^{(1)}(0,\cdot )$; otherwise, we suppose that $u^{(1)}(0,\cdot )=lu^{(2)}(0,\cdot )$ for some $l>0$, with $r({\mathcal {Q}}^{(2)}(\omega ))=1$ the principle eigenvalue with respect to $u^{(2)}(0,\cdot )$. Since $d_6(H)\frac{\partial u^{(1)}}{\partial y}(\omega ,H)+b_1\gamma _Mu^{(1)}(\omega ,H)=0$ and $d_7(H)\frac{\partial u^{(1)}}{\partial y}(\omega ,H)+b_1\gamma _Cu^{(1)}(\omega ,H)=0$, it follows that $d_6(H)\frac{\partial u^{(2)}}{\partial y}(\omega ,H)+b_1\gamma _Mu^{(2)}(\omega ,H)=0$ and $d_7(H)\frac{\partial u^{(2)}}{\partial y}(\omega ,H)+b_1\gamma _Cu^{(2)}(\omega ,H)=0$, which implies that $b_1=b_2$. This leads to a contradiction. Then, from (4.6) we have that $u^{(1)}(0,\cdot )<{\mathcal {Q}}^{(2)}(\omega )u^{(1)}(0,\cdot )$. Finally, from Hess (1991, Theorem 1.7.3) we obtain $r({\mathcal {Q}}^{(2)}(\omega ))>1$, which conradicts $r({\mathcal {Q}}^{(2)}(\omega ))=1$. Therefore, we conclude that $\mu (b_1)>\mu (b_2)$ for $b_1>b_2$. $\square$

## The global dynamics of system (3.1)

To study the global dynamics of system (3.1), we first give the following lemma which will be used later.

### Lemma 5.1

Denote the solution of system (3.1) with initial value $w_0=\psi \in {\textbf {A}}^+$ by $w(t,\cdot ,\psi )$. If there exists some $t^*\ge 0$ such that

$$\begin{aligned} (w_2(t^*,\cdot ,\psi ),w_5(t^*,\cdot ,\psi ),w_6(t^*,\cdot ,\psi ),w_7(t^*,\cdot ,\psi ))\not \equiv 0, \end{aligned}$$

then $w_j(t,y,\psi )>0\ (1\le j\le 7)$ for $(t,y)\in [t^*,\infty )\times [0,H]$. Furthermore, if there exists $\rho ^*>0$ such that $\liminf _{t\rightarrow \infty }w_l(t,\cdot ,\psi )\ge \rho ^*\ (l=2,5,6,7)$, then there exists a $\rho \in (0,\rho ^*)$ such that $\liminf _{t\rightarrow \infty }w_j(t,\cdot ,\psi )\ge \rho$, $1\le j\le 7$.

### Proof

Take $\bar{w}_1(t,y,\psi )$ to be the solution of the following problem

5.1 $$\begin{aligned} {\left\{ \begin{array}{ll} \frac{\partial \bar{w}_1}{\partial t}=\Lambda _1-(\alpha _1(t,y)+\delta _1)\bar{w}_1+\frac{\partial }{\partial y}\left( d_1(y)\frac{\partial \bar{w}_1}{\partial y}\right) ,\quad (t,y)\in (0,\infty )\times (0,H), \\ \frac{\partial \bar{w}_1}{\partial y}(t,0)=\frac{\partial \bar{w}_1}{\partial y}(t,H)=0,\hspace{3.5cm} t > 0,\\ \bar{w}_1(0,y)=\psi _1(y),\hspace{4cm} y\in [0,H] \end{array}\right. } \end{aligned}$$

Applying the comparison principle, one has $w_1(t,y,\psi )\ge \bar{w}_1(t,y,\psi )>0,\ y\in [0,H]$, for all $t>0$. We denote the positive periodic solution of problem (5.1) by $\bar{w}^*_1(t,y)$. Clearly it is globally attractive, and so we obtain

$$\begin{aligned} \liminf _{t\rightarrow \infty }w_1(t,y,\psi )\ge \rho _1:=\min _{(t,y)\in [0,\omega ]\times [0,H]}\bar{w}^*_1(t,y) \end{aligned}$$

for $$y\in [0,H]$$.

Similarly, given the following problem

5.2 $$\begin{aligned} {\left\{ \begin{array}{ll} \frac{\partial \bar{w}_4}{\partial t}=\Lambda _2(t)-(\alpha _2(t,y)+\delta (t))\bar{w}_4+\frac{\partial }{\partial y}\left( d_4(y)\frac{\partial \bar{w}_4}{\partial y}\right) ,\quad (t,y) \in (0,\infty ) \times (0,H),\\ \frac{\partial \bar{w}_4}{\partial y}(t,0)=\frac{\partial \bar{w}_4}{\partial y}(t,H)=0,\hspace{4.5cm} t> 0,\\ \bar{w}_4(0,y)=\psi _4(y),\hspace{5.5cm} y\in [0,H], \end{array}\right. } \end{aligned}$$

we can verify that (5.2) admits a globally attractive positive periodic solution $\bar{w}^*_4(t,y)$, and so we find that

$$\begin{aligned} \liminf _{t\rightarrow \infty }w_3(t,y,\psi )\ge \rho _4:=\min _{(t,y)\in [0,\omega ]\times [0,H]}\bar{w}^*_4(t,y) \end{aligned}$$

for $$y\in [0,H]$$.

Next, we set

$$\begin{aligned} \begin{aligned} f_1(t,y,w_2,w_5,w_6,w_7)&:=w_1\alpha _1(t,y)\frac{w_7}{w_7+\hat{B}(y)}-(\delta _1+\sigma )w_2,\\ f_2(t,y,w_2,w_5,w_6,w_7)&:=w_4\alpha _2(t,y)\frac{w_6}{w_6+\hat{K}(y)}-\delta _2(t)w_5,\\ f_3(t,y,w_2,w_5,w_6,w_7)&:=\exp \left\{ -\int _0^y\frac{\gamma _M}{d_6(x)}dx\right\} \epsilon w_2\\&\quad \,+h_M(t,y)w_6\left( 1-\frac{w_6}{\hat{K}_M(y)}\right) -\delta _Mw_6,\\ f_4(t,y,w_2,w_5,w_6,w_7)&:=\exp \left\{ -\int _0^y\frac{\gamma _C}{d_7(x)}dx\right\} \zeta w_5\\&\quad \,+h_C(t,y)w_7\left( 1-\frac{w_7}{\hat{B}_C(y)}\right) -\delta _Cw_7, \end{aligned} \end{aligned}$$

and let ${\mathbb {F}}(t,y,w_2,w_5,w_6,w_7):=(f_1,f_2,f_3,f_4)$. It follows from the positivity of $w_1$ and $w_4$ that ${\mathbb {F}}$ is irreducible and cooperative (the main diagonal element is negative, and the non main diagonal element is nonnegative). Thus, combining the comparison arguments with $(w_2(t^*,\cdot ,\psi ),w_5(t^*,\cdot ,\psi ),w_6(t^*,\cdot ,\psi ),w_7(t^*,\cdot ,\psi ))\not \equiv 0$, we can verify that

$$\begin{aligned} (w_2(t,\cdot ,\psi ),w_5(t,\cdot ,\psi ),w_6(t,\cdot ,\psi ),w_7(t,\cdot ,\psi ))>0 \end{aligned}$$

for $t>t^*$. Furthermore, for $t>t^*$ we have

$$\begin{aligned} \begin{aligned} \frac{\partial w_3}{\partial t}=&\sigma w_2-\delta _1w_3+\frac{\partial }{\partial y}\left( d_3(y)\frac{\partial w_3}{\partial y}\right) \ge -\delta _1w_3+\frac{\partial }{\partial y}\left( d_3(y)\frac{\partial w_3}{\partial y}\right) , \end{aligned} \end{aligned}$$

and so it is obvious that $w_3(t,y,\psi )>0$ for $(t,y)\in (t^*,\infty )\times [0,H]$. Thus, we can find a large enough $t_0>0$ such that $w_2(t,\cdot ,\psi )>\frac{1}{2}\rho ^*$, and one has

$$\begin{aligned} \frac{\partial w_3}{\partial t}\ge \frac{\sigma \rho ^*}{2} -\delta _1w_3+\frac{\partial }{\partial y}\left( d_3(y)\frac{\partial w_3}{\partial y}\right) . \end{aligned}$$

Therefore, there exists $\rho _3>0$ such that $\liminf _{t\rightarrow \infty }w_3(t,y,\psi )\ge \rho _3$. Setting $\rho =\min \{\rho _1$, $\rho _4$, $\rho _3$, $\rho ^*)$ completes the proof. $\square$

### The global asymptotic stability of the disease-free periodic solution $E_0$

We first state the following Theorem.

#### Theorem 5.2

If ${\mathcal {R}}_0<1$, $d_1(x)=d_2(x)=d_3(x)$ and $d_4(x)=d_5(x)$, then the disease-free periodic solution $E_0=(S^*_1,0,0,\widehat{S}_2(t),0,0,0)$ is globally asymptotically stable.

#### Proof

To complete the proof, we first introduce the following notations to be used later.

$$\begin{aligned} \begin{aligned}&{\mathbb {X}}_0:=\{\psi \in {\textbf {A}}^+;\psi _2,\psi _5,\psi _6,\psi _7\not \equiv 0\},\ {{\mathbb {M}}_0:=\{\psi \in {\textbf {A}}^+;\psi _2=\psi _5=\psi _6=\psi _7\equiv 0\}},\\&\partial {\mathbb {X}}_0:={\textbf {A}}^+\setminus {\mathbb {X}}_0=\{\psi \in {\textbf {A}}^+,\psi _2=0\ \text{ or }\ \psi _5=0\ \text{ or }\ \psi _6=0\ \text{ or }\ \psi _7=0\},\\&{\mathbb {M}}_{\partial }:=\{\psi \in \partial {\mathbb {X}}_0:{\mathcal {P}}^n(\omega )\psi \in \partial {\mathbb {X}}_0,\ n\in {\mathbb {N}}\},\ \text{ where }\ {\mathcal {P}}^n={\mathcal {P}}({\mathcal {P}}^{n-1}), n>1,\ \text{ and }\ {\mathcal {P}}^1={\mathcal {P}},\\&\omega (\psi ):\ \text{ the } \text{ omega } \text{ limit } \text{ set } \text{ of }\ \gamma ^+=\{{\mathcal {Q}}^n(\omega )\psi , n\in {\mathbb {N}}\},\\&{\mathcal {P}}(\omega )(E_0)=E_0. \end{aligned} \end{aligned}$$

From Lemmas 4.1 and 4.2, we know that $r({\mathcal {Q}}(\omega ))<1$ and $\mu =\ln r({\mathcal {Q}}(\omega ))/\omega <0$. Consider the following system with $\kappa >0$

5.3 $$\begin{aligned} {\left\{ \begin{array}{ll} \frac{\partial v^{\kappa }_1}{\partial t}=(S^*_1+\kappa )\alpha _1(t,y)\frac{v^{\kappa }_4}{B}\exp \left\{ \int _0^y\frac{\gamma _C}{d_7(x)}dx\right\} -(\delta _1+\sigma )v^{\kappa }_1+\frac{\partial }{\partial y}\left( d_2(y)\frac{\partial v^{\kappa }_1}{\partial y}\right) ,\\ \frac{\partial v^{\kappa }_2}{\partial t}=(\widehat{S}_2(t,y)+\kappa )\alpha _2(t,y)\frac{v^{\kappa }_3}{K}\exp \left\{ \int _0^y\frac{\gamma _M}{d_6(x)} dx\right\} -\delta _2(t)v^{\kappa }_2+\frac{\partial }{\partial y}\left( d_5(y)\frac{\partial v^{\kappa }_2}{\partial y}\right) ,\\ \frac{\partial v^{\kappa }_3}{\partial t}=\epsilon v^{\kappa }_1\exp \left\{ -\int _0^y\frac{\gamma _M}{d_6(x)}dx\right\} +\gamma _M\frac{\partial v^{\kappa }_3}{\partial y}-\delta _Mv^{\kappa }_3+h_M(t,y)v^{\kappa }_3+\frac{\partial }{\partial y}\left( d_6(y)\frac{\partial v^{\kappa }_3}{\partial y}\right) ,\\ \frac{\partial v^{\kappa }_4}{\partial t}=\zeta v^{\kappa }_2\exp \left\{ -\int _0^y\frac{\gamma _C}{d_7(x)}dx\right\} +\gamma _C\frac{\partial v^{\kappa }_4}{\partial y}-\delta _Cv^{\kappa }_4+h_C(t,y)v^{\kappa }_4+\frac{\partial }{\partial y}\left( d_7(y)\frac{\partial v^{\kappa }_4}{\partial y}\right) ,\\ \frac{\partial v^{\kappa }_i}{\partial y}(t,0)=\frac{\partial v^{\kappa }_i}{\partial y}(t,H)=\frac{\partial v^{\kappa }_k}{\partial y}(t,0)=0,\quad i=1,2,\ k = 3,4,\\ d_6(H)\frac{\partial v^{\kappa }_3}{\partial y}(t,H)=-b_M\gamma _Mv^{\kappa }_3(t,H),\\ d_7(H)\frac{\partial v^{\kappa }_4}{\partial y}(t,H)=-b_C\gamma _Cv^{\kappa }_4(t,H), \end{array}\right. } \end{aligned}$$

for $(t,y)\in [0,\infty )\times (0,H)$. For any $\phi \in {\textbf {Y}}^+$, we denote by $v^{\kappa }(t,y,\phi )=(v^{\kappa }_1,v^{\kappa }_2,v^{\kappa }_3,v^{\kappa }_4)$ to be the solution of system (5.3) with the initial value $v^{\kappa }(0,y,\phi )=\phi (y),y\in [0,H]$. We denote the Poincaré map of system (5.3) by ${\mathcal {Q}}_{\kappa }(\omega ):{\textbf {Y}}\rightarrow {\textbf {Y}}$ and ${\mathcal {Q}}_{\kappa }(\omega )\phi =(v^{\kappa }_1,v^{\kappa }_2,v^{\kappa }_3,v^{\kappa }_4)$ for all $\phi \in {\textbf {Y}}$. Note that since $\lim _{\kappa \rightarrow 0}r({\mathcal {Q}}_{\kappa }(\omega ))=r({\mathcal {Q}}(\omega ))<1$, there exists a small enough constant $\kappa >0$ such that $r({\mathcal {Q}}_{\kappa }(\omega ))<1$. From Lemma 4.2, we can find a solution to system (5.3) of the form $v^{\kappa }(t,\cdot )=e^{\lambda _{\kappa }t}v^*_{\kappa }(t,\cdot )$ where $\lambda _{\kappa }=\ln r({\mathcal {Q}}_{\kappa }(\omega ))/\omega <0$. From (3.5) and system (3.7), we can verify that there exists a $t_1=t_1(\kappa )>0$ such that

5.4 $$\begin{aligned} w_1(t,y)\le S^*_1+\kappa ,\ w_4(t,y)\le \widehat{S}_2(t,y)+\kappa . \end{aligned}$$

Therefore, applying the comparison principle to (5.4) for all $t>t_1$, we have

$$\begin{aligned} {\left\{ \begin{array}{ll} \frac{\partial w_2}{\partial t}\le (S^*_1+\kappa )\alpha _1(t,y)\frac{w_7}{B}\exp \left\{ \int _0^y\frac{\gamma _C}{d_7(x)}dx\right\} -(\delta _1+\sigma )w_2+\frac{\partial }{\partial y}\left( d_2(y)\frac{\partial w_2}{\partial y}\right) ,\\ \frac{\partial w_5}{\partial t}\le (\widehat{S}_2(t,y)+\kappa )\alpha _2(t,y)\frac{w_6}{K}\exp \left\{ \int _0^y\frac{\gamma _M}{d_6(x)}dx\right\} -\delta _2(t)w_5+\frac{\partial }{\partial y}\left( d_5(y)\frac{\partial w_5}{\partial y}\right) ,\\ \frac{\partial w_6}{\partial t}\le \epsilon w_2\exp \left\{ -\int _0^y\frac{\gamma _M}{d_6(x)}dx\right\} +\gamma _M\frac{\partial w_6}{\partial y}-\delta _Mw_6+h_M(t,y)w_6+\frac{\partial }{\partial y}\left( d_6(y)\frac{\partial w_6}{\partial y}\right) ,\\ \frac{\partial w_7}{\partial t}\le \zeta w_5\exp \left\{ -\int _0^y\frac{\gamma _C}{d_7(x)}dx\right\} +\gamma _C\frac{\partial w_7}{\partial y}-\delta _Cw_7+h_C(t,y)w_7+\frac{\partial }{\partial y}\left( d_7(y)\frac{\partial w_7}{\partial y}\right) ,\\ \end{array}\right. } \end{aligned}$$

for $(t,y) \in (t_1,\infty ) \times (0,H)$ with boundary condition

$$\begin{aligned}&\frac{\partial w_i}{\partial y}(t,0)=\frac{\partial w_i}{\partial y}(t,H)=\frac{\partial w_k}{\partial y}(t,0)=0,\quad i=2,5,\ k=6,7,\\&d_6(H)\frac{\partial w_6}{\partial y}(t,H)=-b_M\gamma _Mw_6(t,H),\\&d_7(H)\frac{\partial w_7}{\partial y}(t,H)=-b_C\gamma _Cw_7(t,H), \quad \quad \quad t> t_1. \end{aligned}$$

For $\psi \in {\textbf {A}}^+$, there exists a $n_1>0$ such that

$$\begin{aligned} (w_2(t_1,y,\psi ),w_5(t_1,y,\psi ),w_6(t_1,y,\psi ),w_7(t_1,y,\psi ))\le n_1v^*_{\kappa }(t_1,y),\quad y\in [0,H], \end{aligned}$$

where inequality holds in the component-wise sense. Thus, applying the comparison principle one obtains

$$\begin{aligned} (w_2(t,y,\psi ),w_5(t,y,\psi ),w_6(t,y,\psi ),w_7(t,y,\psi ))\le n_1e^{\lambda _{\kappa }t}v^*_{\kappa }(t,y), \end{aligned}$$

for $(t,y)\in [t_1,\infty )\times [0,H]$. Hence, we can immediately obtain that

$$\begin{aligned} \lim _{t\rightarrow \infty }(w_2(t,y,\psi ),w_5(t,y,\psi ),w_6(t,y,\psi ),w_7(t,y,\psi ))=(0,0,0,0) \end{aligned}$$

uniformly for $y\in [0,H]$ by applying the theory of the limit system. Then, we have $\lim _{t\rightarrow \infty }w_3($ *t*, *y*, $\psi )=0$ uniformly for $y\in [0,H]$. Next, we show $E_0$ is globally attractive in ${\mathbb {M}}_{\partial }$. For this purpose, we first prove that ${\mathbb {M}}_0={\mathbb {M}}_{\partial }$. In fact, it is obvious that ${\mathbb {M}}_0\subset {\mathbb {M}}_{\partial }$. For any $\psi \in {\mathbb {M}}_{\partial }$, it follows from Lemma 5.1 that $(\psi _2(\cdot ),\psi _5(\cdot ),\psi _6(\cdot ),\psi _7(\cdot ))=(0,0,0,0)$ and we can obtain that $\psi \in {\mathbb {M}}_0$, which implies that ${\mathbb {M}}_{\partial }\subset {\mathbb {M}}_0$. Hence, we have that ${\mathbb {M}}_0={\mathbb {M}}_{\partial }$. Based on the above discussion, it is obvious that $w_2(t,\cdot ,\psi )=w_5(t,\cdot ,\psi )=w_6(t,\cdot ,\psi )=w_7(t,\cdot ,\psi )=0$ for $\psi \in {\mathbb {X}}_0$, $t\ge 0$. Then, we obtain that

5.5 $$\begin{aligned} {\left\{ \begin{array}{ll} \frac{\partial w_1}{\partial t}=\Lambda _1-\delta _1w_1+\frac{\partial }{\partial y}\left( d_1(y)\frac{\partial w_1}{\partial y}\right) ,\\ \frac{\partial w_3}{\partial t}=-\delta _1w_3+\frac{\partial }{\partial y}\left( d_3(y)\frac{\partial w_3}{\partial y}\right) ,\\ \frac{\partial w_4}{\partial t}=\Lambda _2(t)-\delta _2(t)w_4+\frac{\partial }{\partial y}\left( d_4(y)\frac{\partial w_4}{\partial y}\right) ,\ (t,y)\in (0,\infty )\times (0,H), \end{array}\right. } \end{aligned}$$

with boundary condition

$$\begin{aligned} \frac{\partial w_i}{\partial y}(t,0)=\frac{\partial w_i}{\partial y}(t,H)=0,\quad i=1,3,4, \quad t>0. \end{aligned}$$

It is obvious that $\lim _{t\rightarrow \infty }w_3(t,y,\psi )=0$ uniformly for $y\in [0,H]$. For system (5.5), applying the theory of internally chain transitive sets (Zhao 2017, Ch. 1), we can immediately obtain that $\lim _{t\rightarrow \infty }w_1(t,y,\psi )=S^*_1$, and that $\lim _{t\rightarrow \infty }w_4(t,y,\psi )=\widehat{S}_2(t,y)$ uniformly for $y\in [0,H]$. It follows that $\omega (\psi )=E_0$, which proves $E_0$ is globally attractive in ${\mathbb {M}}_{\partial }$. Combining Zhao (2017, Lemma 2.2.1) with the fact that system (5.5) is cooperative, the local Lyapunov stability of $E_0$ holds. $\square$

### The uniform persistence of the disease

#### Theorem 5.3

If ${\mathcal {R}}_0>1$, then there exists a constant $\varrho >0$ such that for any $\psi \in {\textbf {A}}^+$ and at least one $\psi _i \not \equiv 0$ for $i=2,5,6,7$, we have

$$\begin{aligned} \liminf \limits _{t\rightarrow \infty }\min \limits _{y\in [0,H]}w_j(t,y,\psi )\ge \varrho ,\quad 1\le j\le 7. \end{aligned}$$

Furthermore, system (3.1) admits at least one positive $\omega$-periodic solution.

#### Proof

Consider the following system with $\varrho >0$

5.6 $$\begin{aligned} {\left\{ \begin{array}{ll} \frac{\partial v^{\varrho }_1}{\partial t}=(S^*_1-\rho )\alpha _1(t,y)\frac{v^{\varrho }_4}{B}\exp \left\{ \int _0^y\frac{\gamma _C}{d_7(x)}dx\right\} -(\delta _1+\sigma )v^{\varrho }_1+\frac{\partial }{\partial y}\left( d_2(y)\frac{\partial v^{\varrho }_1}{\partial y}\right) ,\\ \frac{\partial v^{\varrho }_2}{\partial t}=(\widehat{S}_2(t,y)-\rho )\alpha _2(t,y)\frac{v^{\varrho }_3}{K}\exp \left\{ \int _0^y \frac{\gamma _M}{d_6(x)}dx\right\} -\delta _2(t)v^{\varrho }_2+\frac{\partial }{\partial y}\left( d_5(y)\frac{\partial v^{\varrho }_2}{\partial y}\right) ,\\ \frac{\partial v^{\varrho }_3}{\partial t}=\epsilon v^{\varrho }_1\exp \left\{ -\int _0^y\frac{\gamma _M}{d_6(x)}dx\right\} +\gamma _M\frac{\partial v^{\varrho }_3}{\partial y}-\delta _Mv^{\varrho }_3+h_M(t,y)v^{\varrho }_3\left( 1-\frac{\varrho }{\hat{K}_M}\right) \\ \qquad \qquad +\frac{\partial }{\partial y}\left( d_6(y)\frac{\partial v^{\varrho }_3}{\partial y}\right) ,\\ \frac{\partial v^{\varrho }_4}{\partial t}=\zeta v^{\varrho }_2\exp \left\{ -\int _0^y\frac{\gamma _C}{d_7(x)}dx\right\} +\gamma _C\frac{\partial v^{\varrho }_4}{\partial y}-\delta _Cv^{\varrho }_4+h_C(t,y)v^{\varrho }_4\left( 1-\frac{\varrho }{\hat{B}_C}\right) \\ \qquad \qquad +\frac{\partial }{\partial y}\left( d_7(y)\frac{\partial v^{\varrho }_4}{\partial y}\right) , \end{array}\right. } \end{aligned}$$

for $(t,y)\in [0,\infty )\times (0,H)$ with boundary condition

$$\begin{aligned}&\frac{\partial v^{\varrho }_i}{\partial y}(t,0)=\frac{\partial v^{\varrho }_i}{\partial y}(t,H)=\frac{\partial v^{\varrho }_k}{\partial y}(t,0)=0,\quad i=1,2,\ k=3,4,\\&d_6(H)\frac{\partial v^{\varrho }_3}{\partial y}(t,H)=-b_M\gamma _Mv^{\varrho }_3(t,H),\\&d_7(H)\frac{\partial v^{\varrho }_4}{\partial y}(t,H)=-b_C\gamma _Cv^{\varrho }_4(t,H), \quad \quad \quad t>0. \end{aligned}$$

For any $\phi \in {\textbf {Y}}$, let

$$\begin{aligned} v^{\varrho }(t,y,\phi )=(v^{\varrho }_1(t,y,\phi ),v^{\varrho }_2(t,y,\phi ),v^{\varrho }_3 (t,y,\phi ),v^{\varrho }_4(t,y,\phi )) \end{aligned}$$

be the unique solution of system (5.6) with $v^{\varrho }(t,\cdot ,\phi )=\phi$. Let ${\mathcal {Q}}_{\varrho }(\omega ):{\textbf {Y}}\rightarrow {\textbf {Y}}$ be the Poincaré map of system (5.6) and denote the spectral radius of ${\mathcal {Q}}_{\varrho }(\omega )$ by $r({\mathcal {Q}}_{\varrho }(\omega ))$. Since $\lim _{\varrho \rightarrow 0}r({\mathcal {Q}}_{\varrho }(\omega ))=r({\mathcal {Q}}(\omega ))>1$, we can find a small enough $\varrho >0$ such that $\varrho<S^*_1, \varrho <\widehat{S}_2(t,\cdot )$ and $r({\mathcal {Q}}_{\varrho }(\omega ))>1$. Thus, there exists some $\varrho ^*>0$ such that $\vert \vert {\mathcal {P}}(t)\psi -{\mathcal {P}}(t)E_0\vert \vert <\varrho$ for $t\in [0,\omega ]$, where $\psi$ satisfies $\vert \vert \psi -E_0\vert \vert \le \varrho ^*$.

Now, we prove that

$$\begin{aligned} \limsup \limits _{n\rightarrow \infty }\vert \vert {\mathcal {P}}^n(\omega )(\psi )-E_0\vert \vert \ge \varrho ^*,\ \psi \in {\mathbb {X}}_0. \end{aligned}$$

If it is not true, then there exists $n_1\ge 1$ such that $\vert \vert {\mathcal {P}}^n(\omega )(\psi _0)-E_0\vert \vert <\varrho ^*$ for $n\ge n_1$ and $t\ge n_1\omega$. Setting $\tilde{t}=t-n\omega$ with $\tilde{t}\in [0,\omega )$ and $n=[t/\omega ]$ yields

5.7 $$\begin{aligned} \vert \vert {\mathcal {P}}(t)\psi _0-{\mathcal {P}}(t)E_0\vert \vert =\vert \vert {\mathcal {P}} (\tilde{t})({\mathcal {P}}(\omega )\psi _0)-{\mathcal {P}}(\tilde{t})E_0\vert \vert <\varrho . \end{aligned}$$

From Lemma 5.1 and (5.7), we have

$$\begin{aligned}&w_1(t,y,\psi _0)>S^*_1-\varrho , \\&w_4(t,y,\psi _0)>\widehat{S}_2(t,y)-\varrho , \\&0<w_i(t,y,\psi _0)<\varrho ,\quad i = 2,3,5,6,7, \end{aligned}$$

for $t\ge n_1\omega ,\ y\in [0,H]$. Thus, we have

$$\begin{aligned} {\left\{ \begin{array}{ll} \frac{\partial w_2}{\partial t}\ge (S^*_1-\varrho )\alpha _1(t,y)\frac{w_7}{B}\exp \left\{ \int _0^y\frac{\gamma _C}{d_7(x)}dx\right\} -(\delta _1+\sigma )w_2+\frac{\partial }{\partial y}\left( d_2(y)\frac{\partial w_2}{\partial y}\right) ,\\ \frac{\partial w_5}{\partial t}\ge (\widehat{S}_2(t,y)-\varrho )\alpha _2(t,y)\frac{w_6}{K}\exp \left\{ \int _0^y\frac{\gamma _M}{d_6(x)}dx\right\} -\delta _2(t)w_5+\frac{\partial }{\partial y}\left( d_5(y)\frac{\partial w_5}{\partial y}\right) ,\\ \frac{\partial w_6}{\partial t}\ge \epsilon w_2\exp \left\{ -\int _0^y\frac{\gamma _M}{d_6(x)}dx\right\} +\gamma _M\frac{\partial w_6}{\partial y}-\delta _Mw_6+h_M(t,y)w_6\left( 1-\frac{\varrho }{\hat{K}_M}\right) \\ \qquad \qquad +\frac{\partial }{\partial y}\left( d_6(y)\frac{\partial w_6}{\partial y}\right) ,\\ \frac{\partial w_7}{\partial t}\ge \zeta w_5\exp \left\{ -\int _0^y\frac{\gamma _C}{d_7(x)}dx\right\} +\gamma _C\frac{\partial w_7}{\partial y}-\delta _Cw_7+h_C(t,y)w_7\left( 1-\frac{\varrho }{\hat{B}_C}\right) \\ \qquad \qquad +\frac{\partial }{\partial y}\left( d_7(y)\frac{\partial w_7}{\partial y}\right) , \end{array}\right. } \end{aligned}$$

for $(t,y) \in (n_1 \omega , \infty ) \times (0,H)$ with boundary condition

$$\begin{aligned}&\frac{\partial w_i}{\partial y}(t,0)=\frac{\partial w_i}{\partial y}(t,H)=\frac{\partial w_k}{\partial y}(t,0)=0,\quad i=2,5,\ k=6,7,\\&d_6(H)\frac{\partial w_6}{\partial y}(t,H)=-b_M\gamma _Mw_6(t,H),\\&d_7(H)\frac{\partial w_7}{\partial y}(t,H)=-b_C\gamma _Cw_7(t,H), \quad \quad \quad t > n_1 \omega . \end{aligned}$$

Since $(w_2(t,y,\psi _0),w_5(t,y,\psi _0),w_6(t,y,\psi _0),w_7(t,y,\psi _0))>0$ for $(t,y)\in [0,\infty )\times [0,H]$, it follows that there exists a constant $n_2>0$ such that

$$\begin{aligned} (w_2(n_2\omega ,y,\psi _0),w_5(n_2\omega ,y,\psi _0),w_6(n_2\omega ,y,\psi _0),w_7(n_2\omega ,y,\psi _0))\ge n_2v^{\varrho }(n_2\omega ,y) \end{aligned}$$

for $y\in [0,H]$, where $v^{\varrho }(n_2\omega ,y)=e^{\lambda _{\varrho }t}v^*_{\varrho }(t,y)$, and $v^*_{\varrho }(t,y)$ is a positive $\omega$-periodic function associated with the solution $v^{\varrho }(n_2\omega ,y)$ of system (5.6) with $\lambda _{\varrho }=\ln r({\mathcal {Q}}_{\varrho }(\omega ))/\omega$. Hence, we have

$$\begin{aligned} (w_2(t,y,\psi _0),w_5(t,y,\psi _0),w_6(t,y,\psi _0),w_7(t,y,\psi _0))> n_2e^{\lambda _{\varrho }t}v^*_{\varrho }(t,y) \end{aligned}$$

for $(t,y)\in [n_2\omega ,\infty )\times [0,H]$. It follows that

$$\begin{aligned} \lim _{t\rightarrow \infty }(w_2(t,y,\psi _0),w_5(t,y,\psi _0),w_6(t,y,\psi _0),w_7(t,y,\psi _0))=\infty , \end{aligned}$$

which leads to a contradiction with the ultimately boundedness of system (3.1).

From Theorem 5.2, we know that there is no cycle in ${\mathbb {M}}_{\partial }$ from $E_0$ to $E_0$, $\limsup _{n\rightarrow \infty }\vert \vert {\mathcal {P}}^n(\omega )(\psi )-E_0\vert \vert \ge \varrho ^*,\ \psi \in {\mathbb {X}}_0$ implies that $E_0$ is an isolated invariant set for ${\mathcal {P}}(\omega )$ in ${\textbf {A}}^+$, and the intersection of the stable set of $E_0$ for ${\mathcal {P}}(\omega ), W^s(E_0)$ and ${\mathbb {X}}_0$ is an empty set (i.e., $W^s (E_0)\cap {\mathbb {X}}_0=\emptyset )$. From Zhao (2017, Theorem 1.3.1), the uniform persistence of ${\mathcal {P}}(\omega )$ holds. Furthermore, it follows from Magal and Zhao (2005, Theorem 4.5) that there exists a global attractor $A_0$ associated with ${\mathcal {P}}(\omega ): {\mathbb {X}}_0\rightarrow {\mathbb {X}}_0$ and there exists a fixed point $\psi ^*$ of ${\mathcal {P}}(\omega )$ in ${\mathbb {X}}_0$. Based on Lemma 5.1, it is obvious that $w(t,y,\psi )$ is a strictly positive $\omega$-periodic solution of system (3.1). It follows from ${\mathcal {P}}(\omega )A_0=A_0$ that $\psi _2(\cdot )>0,\psi _5(\cdot )>0,\psi _6(\cdot )>0$ and $\psi _7(\cdot )>0$ for $\psi \in {\mathbb {X}}_0$. Set ${\mathbb {G}}:=\cup _{t\in [0,\omega ]}{\mathcal {P}}(t)A_0$. Then, we have ${\mathbb {G}}\subset {\mathbb {X}}_0$ and the limits of the norm-induced distance $\lim _{t\rightarrow \infty }\text{ d }({\mathcal {P}}(t),{\mathbb {G}})=0$ for $\psi \in {\mathbb {X}}_0$. Denote by $q:{\textbf {A}}^+\rightarrow {\mathbb {R}}_+$ the following

$$\begin{aligned} q(\psi )=\min \{\min _{y\in \overline{\Omega }}\psi _2(y),\min _{y\in \overline{\Omega }} \psi _5(y),\min _{y\in \overline{\Omega }}\psi _6(y),\min _{y\in \overline{\Omega }}\psi _7(y)\} \end{aligned}$$

for $\psi \in {\textbf {A}}^+$. We can obtain that $\inf _{\psi \in {\mathbb {G}}}q(\psi )=\min _{\psi \in {\mathbb {G}}}q(\psi )>0$ from ${\mathbb {G}}$ is a compact subset of ${\mathbb {X}}_0$. Finally, we can verify that there exists a $\varrho ^*>0$ such that

$$\begin{aligned} \liminf \limits _{t\rightarrow \infty }q({\mathcal {P}}(t)\psi )=\liminf \limits _{t\rightarrow \infty } \min _{i=2,5,6,7}\left\{ \min _{y\in \overline{\Omega }}\psi _i(t,y,\psi )\right\} \ge \varrho ^*. \end{aligned}$$

Hence, based on Lemma 5.1, there admits an $\varrho \in (0,\varrho ^*)$ such that

$$\begin{aligned} \liminf _{t\rightarrow \infty }\min _{y\in \overline{\Omega }}w_j(t,y,\psi )\ge \varrho \end{aligned}$$

for all $\psi \in {\mathbb {X}}_0$. For $\psi \in {\textbf {A}}^+$ with $\psi _i \not \equiv 0$ for some $i=2,5,6,7$, it is easy to see that $\psi \notin {\mathbb {M}}_0$. Hence, we can verify that there exists some $n^*=n^*(\psi )\ge 0$ such that ${\mathcal {P}}^{n^*}(\omega )\psi \in {\mathbb {X}}_0$. If this were not the case, ${\mathcal {P}}^n(\omega )\psi \in \partial {\mathbb {X}}_0$, and we therefore obtain $\psi \in {\mathbb {M}}_0$, which leads to a contradiction. Note that since ${\mathcal {P}}(t)\psi ={\mathcal {P}}(t-n^*\omega )({\mathcal {P}}^{n^*}(\omega )\psi ), t)\ge n^*\omega$, we immediately find that $\liminf _{t\rightarrow \infty }\min \limits _{y\in \overline{\Omega }}w_j(t,y,\psi )\ge \varrho$ for $1\le j\le 7$. $\square$

## Numerical simulation

In this section we explore some numerical simulations to compliment the preceding analysis. In particular, we are interested in the influence of heterogeneity of the environment and advection speeds on the transmission of schistosomiasis. For simplicity, we choose

$$\begin{aligned} H=3\pi ,\ d_1(y)=0.8,\ d_2(y)=0.2,\ d_3(y)=0.1,\\ d_4(y)=0.3,\ d_5(y)=0.6,\ d_6(y)=0.1,\ d_7(y)=0.2, \end{aligned}$$

and the initial values are chosen to be

$$\begin{aligned}&S_{10}(y)=S_{10}(1-0.3\cos (2\pi y)),\ S_{20}(y)=S_{20}(1-0.3\cos (2\pi y)),\\&I_{10}(y)=I_{10}(1-0.3\cos (2\pi y)),\ I_{20}(y)=I_{20}(1-0.3\cos (2\pi y)),\\&R_{0}(y)=R_{0}(1-0.3\cos (2\pi y)),\ M_{0}(y)=M_{0}(1-0.3\cos (2\pi y)),\\&C_{0}(y)=C_{0}(1-0.3\cos (2\pi y)), \end{aligned}$$

where

$$\begin{aligned}&(S_{10},S_{20},I_{10},I_{20},R_0,M_0,C_0)\\&\quad =(3.173\times 20^{7}, 124, 30, 7.547\times 10^5, 2.632\times 10^5, 25916 2.05\times 10^7). \end{aligned}$$

These parameter ranges for the initial data are based upon the reported data found in Li et al. (2017). For estimating diffusion rates, acquiring precise values or even a suitable order of magnitude for each population poses a significant challenge. Consequently, the chosen rates, though somewhat arbitrary, are primarily aimed at providing a more insightful representation of the analytical findings.

### Setup of the finite difference method

In this subsection, we apply the finite difference method to achieve discretization of model (3.1) in one-dimensional space. This numerical method has been used in many PDE infectious disease model studies (Tian and Ruan 2018; Wang et al. 2022), and convergence and accuracy can be guaranteed. According to Table 2, we can rewrite $w_j(t,y)\ (i=1,2,\ldots ,5)$ equations model (3.1) as follows

6.1 $$\begin{aligned} \left\{ \begin{aligned}&u_{t}=du_{yy}+f(u,v), \quad y\in [0,H],t>0,\\&u(0,y)=u_0(y)\ge 0, \quad y\in [0,H],\\&u_y(t,0)=u_y(t,H)=0, \quad t>0, \end{aligned} \right. \end{aligned}$$

where $$u=(w_1,w_2,w_3,w_4,w_5)^T,v=(w_6,w_7)^T, d=(d_1,d_2,d_3,d_4,d_5)^T$$ and

$$\begin{aligned} f(u,v)= \begin{pmatrix} \Lambda _1-\alpha _1(t,y)\frac{w_1w_7}{w_7+B(y)}-\delta _1w_1 \\ \alpha _1(t,y)\frac{w_1w_7}{w_7+\hat{B}(y)}-(\delta _1+\sigma )w_2 \\ \sigma w_2-\delta _1w_3 \\ \Lambda _2(t)-\alpha _2(t,y)\frac{w_4w_6}{w_6+\hat{K}(y)}-\delta (t)w_4 \\ \alpha _2(t,y)\frac{w_4w_6}{w_6+\hat{K}(y)}-\delta _2(t)w_5 \end{pmatrix} \end{aligned}$$

We divide the interval [0, *H*] into *n* equally sized sub-intervals $[y^i,y^{i+1}],i=1,\ldots ,n+1$ with $y^i=y^{i-1}+\Delta y,y^1=0,y^{n+1}=L$ and $\Delta y=H/n$. We also denote the step size by $\Delta t=T_{max}/m, j=1,\ldots ,m$. Set $u^i_j=u(t_j,y^i)$, $t+j=t_{j-1}+\Delta t$, $y^i=y^{i-1}+\Delta y$, $1\le j\le m$, $1\le i\le n+1$. We use backward and central differences to discretize $u_t,u_y$ and $u_{yy}$ as follows:

$$\begin{aligned} u_t(t_j,y^i)=\frac{u^i_j-u^{i}_{j-1}}{\Delta t},u_y(t_j,y^i)=\frac{u^i_j-u^{i-1}_j}{\Delta y}, u_{yy}(t_j,y^i)=\frac{u^{i+1}_j-2u^i_j+u^{i-1}_j}{(\Delta y)^2}. \end{aligned}$$

**Table 2:** The value of the parameters of system (2.1)

Parameters	Value	Unit	Source
$\Lambda _1$	$2.431\times 10^4$	month^-1^	Li et al. (2017)
$\Lambda _2$	$5.660\times 10^5$	month^-1^	Li et al. (2017)
$\alpha _1(t,y)$	$\alpha _1(1+a_1\sin (c_1+\frac{\pi t}{6})(\tilde{a}_1+\tilde{c}_1\cos (2\pi y))$	month^-1^	Li et al. (2017), Wang et al. (2022)
$\alpha _2(t,y)$	$\alpha _2(1+a_2\sin (c_2+\frac{\pi t}{6})(\tilde{a}_2+\tilde{c}_2\cos (2\pi y))$	month^-1^	Li et al. (2017), Wang et al. (2022)
$\delta _1$	$1.126\times 10^{-3}$	month^-1^	Zhang and Zhao (2020)
$\delta _2(t)$	$1.788\times 10^{-2}$	month^-1^	Zhang and Zhao (2020)
$d_j(x)=d_j (1\le j\le 7)$	–	km^2^month^-1^	Varied
$\delta _M$	27	month^-1^	Li et al. (2017), Zhang and Zhao (2020)
$\delta _C$	0.12	month^-1^	Li et al. (2017), Zhang and Zhao (2020)
$\sigma$	0.02	month^-1^	Varied
$\epsilon$	209	month^-1^	Li et al. (2017), Zhang and Zhao (2020)
$\zeta$	78	month^-1^	Li et al. (2017)
$\gamma _M$	–	km month^-1^	Varied
$\gamma _C$	–	km month^-1^	Varied
$b_M$	2	–	Estimated
$b_C$	2	–	Estimated
*K*(*B*)	$10^{5}(2\times 10^{5})$	cells ml^-1^	Wang et al. (2022)
$K_M(B_C)$	$2\times 10^{6}(3\times 10^{6})$	cells ml^-1^	Wang et al. (2022)
$h_l(t,y)(l=M,C)$	$h_{1l}+h_{2l}\sin (\pi t/6)$	month^-1^	Wang et al. (2022)

Thus, for $w_j(t,y)$ in model (6.1), we have

$$\begin{aligned} \left\{ \begin{aligned}&\frac{u^i_j-u^i_{j-1}}{\Delta t}=d\frac{u^{i+1}_j-2u^i_j+u^{i-1}_j}{(\Delta y)^2}+f(u^i_j),\\&u^i_{j-1}=-ru^{i+1}_j+(1+2r)u^i_j-ru^{i-1}_j-\Delta t\cdot f(u^i_j,v^i_j), r=\frac{d\Delta t}{(\Delta y)^2}, 2\le i\le n. \end{aligned} \right. \end{aligned}$$

For the boundary points $i=1$ and $i=n+1$, since model (3.1) satisfies the Neumann boundary condition, it follows that $u_y(0)=0$, $u_y(t_j,0)=\frac{u^1_j-u^0_j}{\Delta y}=0$, so we have $u^0_j=u^1_j$. Then, $u^1_{j-1}=-ru^2_j+(1+2r)u^1_j-ru^0_j-\Delta t\cdot f(u^1_j,v^1_j)=-ru^2_j+(1+r)u^1_j-\Delta t \cdot f(u^1_j,v^1_j)$. Also $u^{n+2}_j=u^{n+1}_j$, so $u^{n+1}_{j-1}=-ru^{n+2}_j+(1+2r)u^{n+1}_j-ru^n_j-\Delta t\cdot f(u^{n+1}_j,v^{n+1}_j)=(1+r)u^{n+1}_j-ru^n_j-\Delta t\cdot f(u^{n+1}_j,v^{n+1}_j)$.

Similarly, we can rewrite $w_6(t,y)$ and $w_7(t,y)$ in model (3.1) as follows

6.2 $$\begin{aligned} \left\{ \begin{aligned}&v_{t}=dv_{yy}+\gamma v_x+g(v,u), \quad y\in [0,H],t>0,\\&v(0,y)=v_0(y)\ge 0, \quad y\in [0,H],\\&dv_y(t,0)=0,dv_y(t,H)-\gamma v(t,H)=-b\gamma v(t,H), \quad t>0, \end{aligned} \right. \end{aligned}$$

where $$v=(w_6,w_7)^T,\gamma =(\gamma _C,\gamma _M)^T,b=(b_C,b_M)^T,d=(d_6,d_7)^T$$ and

$$\begin{aligned} g(v)= \begin{pmatrix} \epsilon w_2\exp \left\{ -\int _0^y\frac{\gamma _M}{d_6(x)}dx\right\} + h_M(t,y)w_6\left( 1-\frac{w_6}{\hat{K}_M(y)}\right) -\delta _M(t)w_6 \\ \zeta w_5\exp \left\{ -\int _0^y\frac{\gamma _C}{d_7(x)}dx\right\} +h_C(t,y)w_7 \left( 1-\frac{w_7}{\hat{B}_C(y)}\right) -\delta _C(t)w_7. \end{pmatrix} \end{aligned}$$

Thus, we can obtain

$$\begin{aligned} \left\{ \begin{aligned}&\frac{v^i_j-v^i_{j-1}}{\Delta t}=d\frac{v^{i+1}_j-2v^i_j+v^{i-1}_j}{(\Delta y)^2}+\gamma \frac{v^i_j-v^{i-1}_j}{\Delta y}+g(v^i_j,u^i_j)\\&v^i_{j-1}=-rv^{i+1}_j+(1+2r-s)v^i_j-(r-s)v^{i-1}_j-\Delta t\cdot g(u^i_j,v^i_j), s=\frac{\gamma \Delta t}{\Delta y},2\le i\le n. \end{aligned} \right. \end{aligned}$$

As argued previously, since model (6.2) satisfies Neumann and no-flux boundary conditions, it follows that $dv_y(t,0)=0$, and $v^0_j=v^1_j$. Thus, we have $v^1_{j-1}=-rv^2_j+(1+r-s)u^1_j-\Delta t\cdot g(u^1_j,v^1_j)$. For $dv_y(t,H)=\gamma (1-b)v(t,H)$, we have $v^{n+2}_j=\left( 1-\frac{\gamma (1-b) \Delta y}{d}\right) v^{n+1}_j=(1-\frac{(1-b)s}{r})v^{n+1}_j$. Thus, we obtain $v^{n+1}_{j-1}=(1+r+(2-b)s)u^{n+1}_j-(r-s)v^n_j-\Delta t\cdot g(u^{n+1}_j,v^{n+1}_j)$.

### The dynamical behavior of system (3.1)

We apply the numerical scheme mentioned in Wu et al. (2022). Many of the parameters included in the model can be obtained via measurement while others are estimated. Similar to Li et al. (2017), Wang et al. (2022), we take $\alpha _1(t,y)=\alpha _1(1+a_1\sin (c_1+\frac{\pi t}{6})(\tilde{a}_1+\tilde{c}_1\cos (2\pi y))$ and $\alpha _2(t,y)=\alpha _2(1+a_2\sin (c_2+\frac{\pi t}{6})(\tilde{a}_2+\tilde{c}_2\cos (2\pi y))$, and set $\alpha _1=8\times 10^{-14}$, $\alpha _2=1.974\times 10^{-8}$ (Li et al. 2017), $a_1=a_2=0.85, \tilde{a}_1=\tilde{a}_2=0.5, \tilde{c}_1=\tilde{c}_2=0.8$. We set $h_l(t,y)(l=M,C)=h_{1l}+h_{2l}\sin (\pi t/6)$ with $h_{1l}=h_{2l}=0.5$. We choose $\gamma _M=1$, $\gamma _C=2$; other parameter values are taken from Zhang and Zhao (2020) and can be found in Table 2.

Note that Lemma 4.3 provides us a method to calculate the value of ${\mathcal {R}}_0$ numerically. To this end, set $\varphi \in \text{ Int }({\textbf {Y}}^+)$ and denote by

$$\begin{aligned} g_m=\vert \vert W(\omega ,0,\mu )\varphi _{m-1}\vert \vert _{{\textbf {Y}}}, \\ \varphi _m=\frac{W(\omega ,0,\mu )\varphi _{n-1}}{g_n}, \end{aligned}$$

for $m\ge 1$ and any $\mu \in (0,\infty )$. Here $\{W(t,s,\mu ):t\ge s\}$ is the family of evolution operators ${\mathcal {F}}$ and ${\mathcal {V}}$ associated with the linear equation

$$\begin{aligned} {\left\{ \begin{array}{ll} \frac{\partial w}{\partial t}=-{\mathcal {V}}w+\mu {\mathcal {F}}(x,t)w,\quad (t,y) \in (0,\infty ) \times (0,H),\\ {\mathbb {B}}_bw=0,\hspace{3.5cm} y=0,H,\quad t>0. \end{array}\right. } \end{aligned}$$

**Fig. 2:**
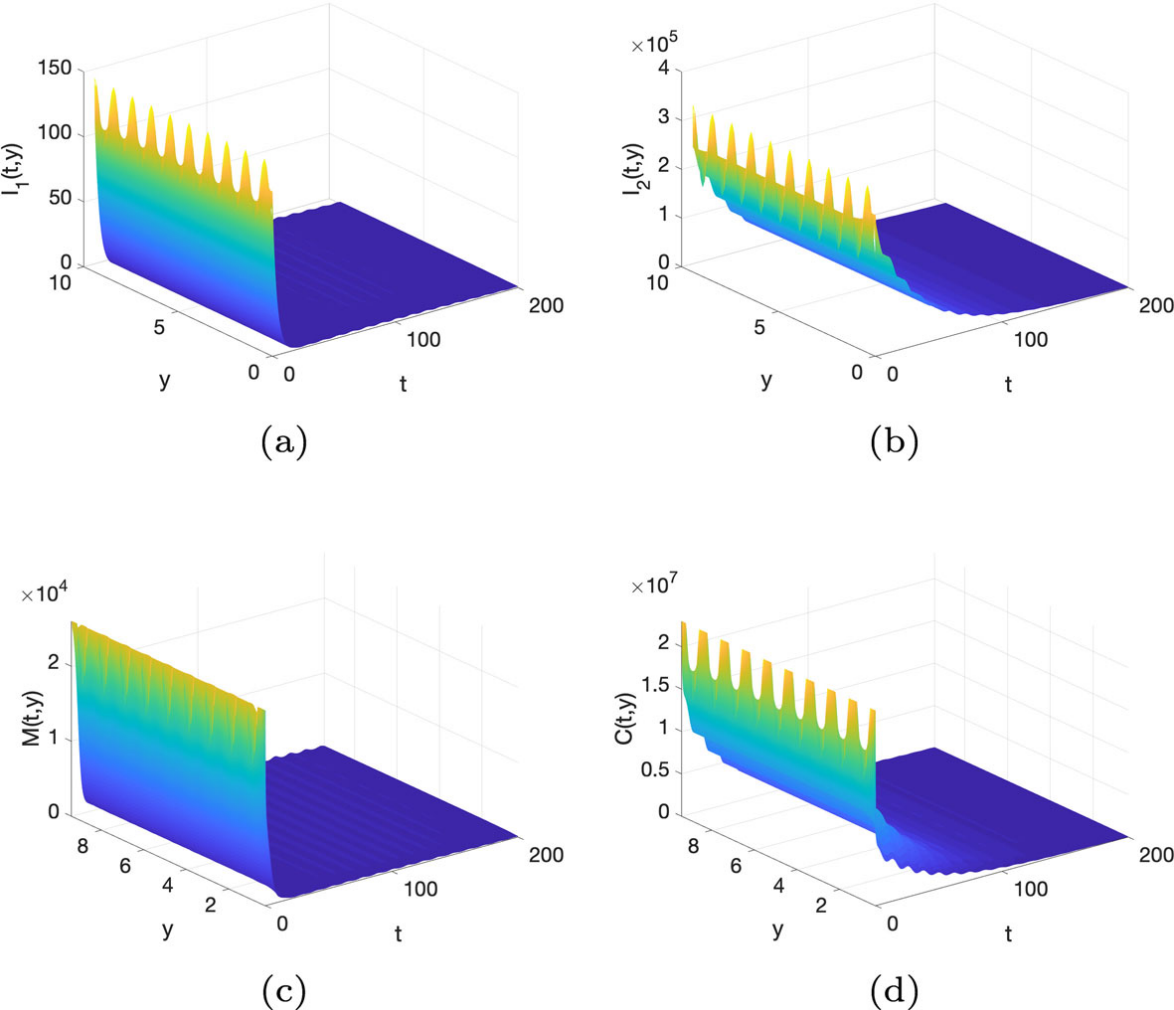
The dynamical behavior of system (2.1) when ${\mathcal {R}}_0<1$

**Fig. 3:**
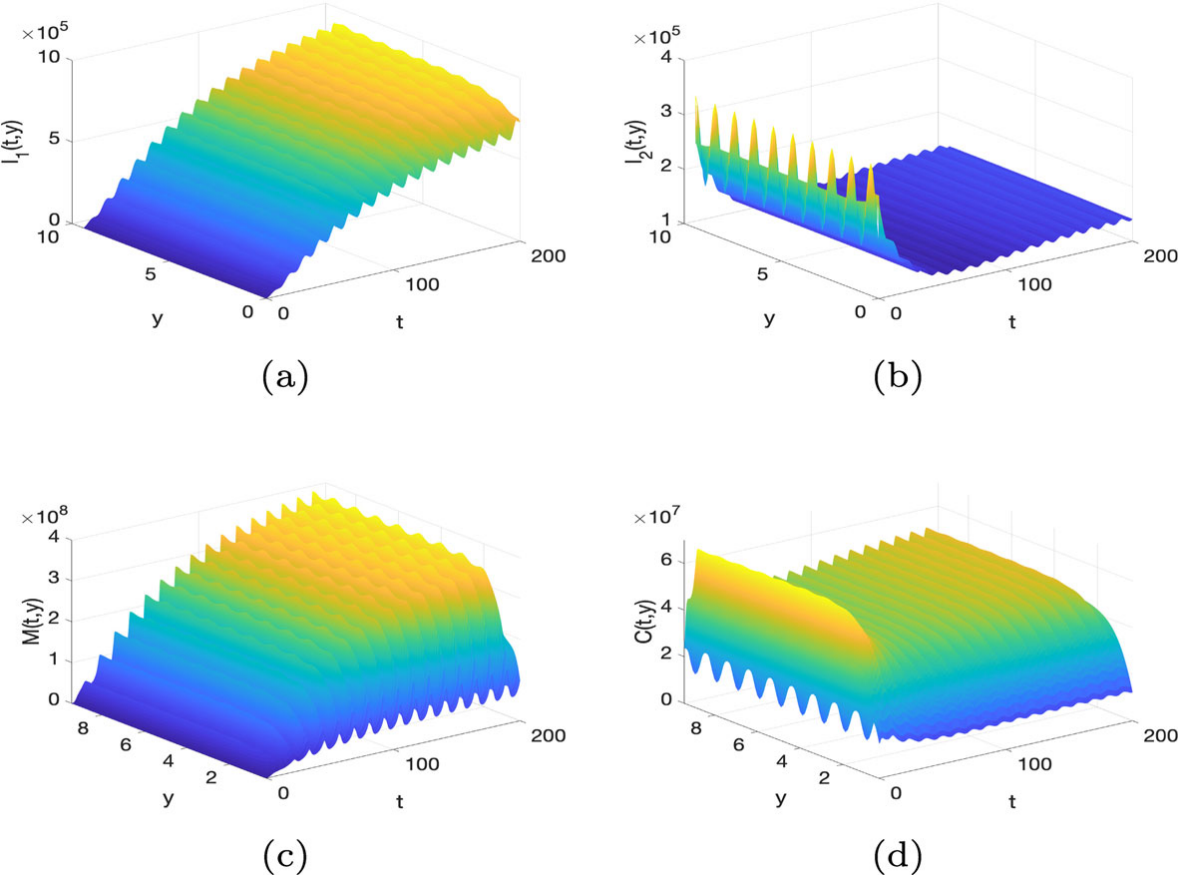
The dynamical behavior of system (2.1) when ${\mathcal {R}}_0>1$

Based on Wu et al. (2022, Lemma 2.5), we know that $r(W(\omega ,0,\mu ))=\lim _{m\rightarrow \infty }g_m$ whenever the limit exists. Then, we use that ${\mathcal {R}}_0=1/\mu$. Utilizing the MATLAB software, we find that ${\mathcal {R}}_0=0.7912<1$, which implies that schistosomiasis transmission cannot be sustained (see Fig. 2), which aligns with the theoretical result of Theorem 5.2. When we choose $\alpha _1=8\times 10^{-14}$, $\alpha _2=1.974\times 10^{-8}$ and $\gamma _M=0.1$, $\gamma _C=0.2$ and all other parameter values as in Fig. 2. We then find that ${\mathcal {R}}_0=2.518>1$ and so schistosomiasis and miracidia(cercariae) prevalence oscillates in both river and human hosts. That means that schistosomiasis will persist in both river and human hosts (see Fig. 3), which aligns with the theoretical result of Theorem 5.3.

### The impact of advection, spatial heterogeneity and seasonality on the schistosomiasis transmission

**Fig. 4:**
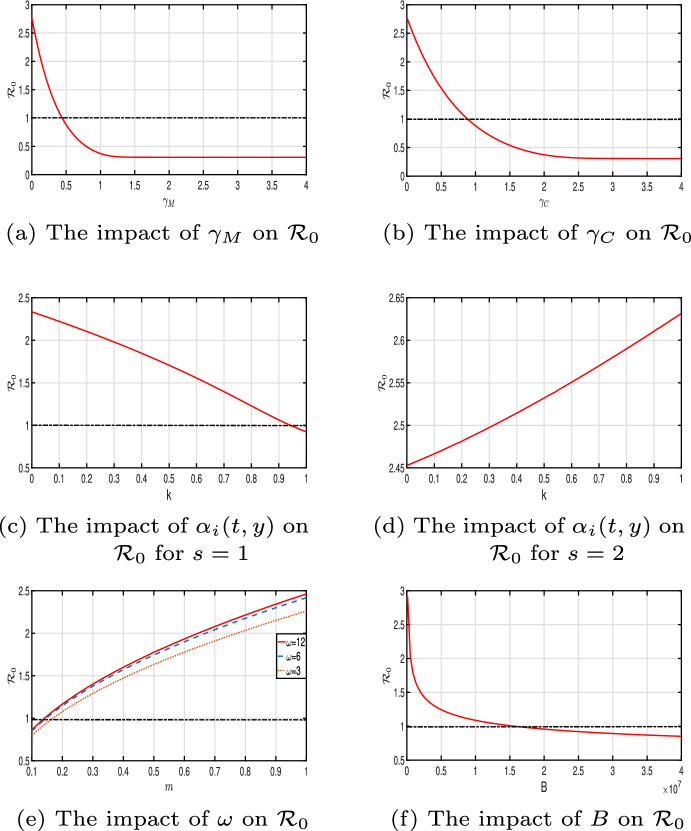
The impact of advection, spatial heterogeneity and seasonality on ${\mathcal {R}}_0$. The values of the other parameters are the same as in Fig. 3

To study the impact of advection on the schistosomiasis spread, similar to the numerical method found in Aatif et al. (2023), we examine the influence of the advection rates $\gamma _M$ and $\gamma _C$ on the value of ${\mathcal {R}}_0$ in Fig. 4a and b, respectively. It can be seen that ${\mathcal {R}}_0$ decreases sharply with $\gamma _M$ ranging from 0 to 1 and with $\gamma _C$ ranging from 0 to 2, respectively. The value of ${\mathcal {R}}_0$ goes down slowly as $\gamma _M$ and $\gamma _C$ continue to increase to 4. This indicates that a faster flow rate of the river could lower the risk of a schistosomiasis outbreak. The corresponding impact of advection rates of miracidia and cercariae on the dynamical behavior of system (3.1) is shown in Fig. 6.

We are also interested in how spatial heterogeneity of the transmission rates from cercariae to humans and from miracidia to snails affects the value of ${\mathcal {R}}_0$. To this end, we assume $\alpha _i(t,y)=\alpha _iF_i(y)\cdot T_i(t)\ (i=1,2)$, where $T_i(t)=1+0.5\sin (0.25+\frac{\pi t}{6})$, and $F_i(y)=0.85+0.8\cos (2\pi y)$. To study the influence of *F*(*y*) on ${\mathcal {R}}_0$, we set $F_i(y;k;s)=0.85+k0.8\cos (s\pi y)$ with $k\in [0,1]$, $s=1,2$. Figure 4c and d show the changes in the spatial dependence of $\alpha _i(t,y)$ decreases and increases the risk of the disease outbreak for $s=1$ and $s=2$, respectively. We set $T_i(t)=m(1+0.5\sin (0.25+\frac{2\pi t}{\omega }))$ to study the impact of seasonality on the value of ${\mathcal {R}}_0$ in Fig. 4. In Fig. 4e, the influence of seasonality is shown by comparing the value of ${\mathcal {R}}_0$ under annual infection $\omega =12$, semi-annual infection $\omega =6$ and quarter-infection $\omega =3$, respectively. It is obvious that the disease risk ${\mathcal {R}}_0$ is an increasing function with respect to *m* and the risk of the disease outbreak under annual infections is apparently higher than in quarter-infection and semi-infection cases. This indicates that seasonality plays a key role in the transmission of schistosomiasis. We also examine the impact of half saturation rate *B* of cercariae on the disease risk ${\mathcal {R}}_0$ in Fig. 4d. As expected from the form appearing in the model, we observe that ${\mathcal {R}}_0$ decreases as *B* increases (i.e., as *B* increases the infection rate decreases), which implies the saturation of infection function also has a certain impact on the spread of disease: this value could increase or decrease depending on the level of resistance to infection in the human population or the parasite’s ability to infect the human population.

### Sensitivity analysis

The main objective of this subsection is to discuss the sensitivity of ${\mathcal {R}}_0$. For this purpose, we apply the normalized forward sensitivity index and the central difference approximation to evaluate the derivatives (Chitnis et al. 2008; Kong et al. 2018; Wang et al. 2019) as follows:

6.3 $$\begin{aligned} \begin{aligned}&\text{ Sensitivity } \text{ index(S.I.) }=\frac{\partial {\mathcal {R}}_0}{\partial (\text{ parameter})}\cdot \frac{\text{ parameter }}{{\mathcal {R}}_0},\\&\frac{\partial {\mathcal {R}}_0}{\partial (\text{ parameter})}=\frac{{\mathcal {R}}_0(\text{ parameter }+h) -{\mathcal {R}}_0(\text{ parameter }-h)}{2h}+O(h^2). \end{aligned} \end{aligned}$$

Setting $h=1\%$ of the parameter value, Equation (6.3) becomes

$$\begin{aligned} \text{ S.I. }=\frac{{\mathcal {R}}_0(1.01P)-{\mathcal {R}}_0(0.99P)}{0.02{\mathcal {R}}_0(P)}. \end{aligned}$$

**Fig. 5:**
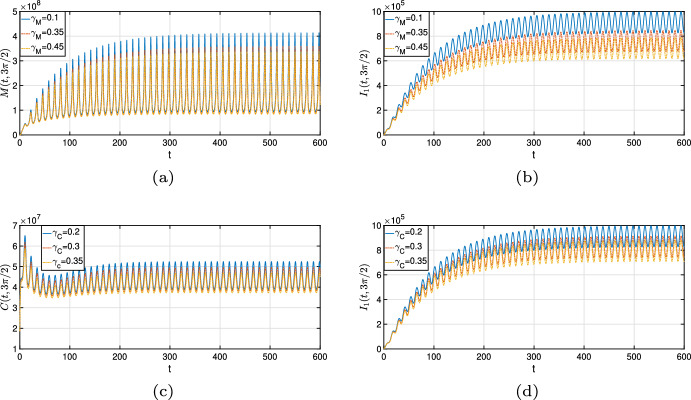
The impact of advection on the dynamical behavior of the solution of system (3.1). The values of the other parameters are the same as in Fig. 3

Using this formula we obtain that the sensitivity indices of ${\mathcal {R}}_0$ to parameters $\gamma _M$, $\gamma _C$, $\lambda _1$, $\Lambda _2$, $\sigma$, $\epsilon$, $\zeta$, $\alpha _1$, $\alpha _2$, $\delta _1$, $\delta _2$, $\delta _M$ and $\delta _C$ are, respectively, $-0.6432$, $-0.6066$, 1.033, 0.844, $-0.3008$, 0.2345, 0.4091, 0.6908, 0.5778, $-0.3806$, $-0.2088$, $-0.233$, and $-0.344$ (see Fig. 5). In Fig. 5, we fixed the parameter values when we calculate the sensitivity indices. It can be seen that the parameters $\Lambda _1,\Lambda _2,\epsilon ,\zeta ,\alpha _1,\alpha _2$ are positively correlated with ${\mathcal {R}}_0$, while $\gamma _M,\gamma _C,\sigma ,\delta _1,\delta _2,\delta _M,\delta _C$ are negatively correlated with ${\mathcal {R}}_0$. This indicates that increasing the recruitment rate of sea snails, the shedding rate of parasitic eggs and the releasing rate of cercariae from snails will increase the value of the basic reproduction number ${\mathcal {R}}_0$, while increasing the mortality rate of sea snails, as well as the mortality rate of larvae and cercariae, will reduce the value of the basic reproduction number. In practical terms, the removal of snails, larvae, and cercariae in rivers will effectively reduce the outbreak of diseases, which is actually one of the most effective methods commonly adopted for schistosomiasis prevention and control. Of particular interest is the relatively strong negative correlation with the advection rates $\gamma _M$ and $\gamma _C$: given the option, it may be advisable to engage in bathing/cleaning activities in areas of the river with a moderate flow rate as opposed to areas of still water. This is a simple and cost effective approach when compared to other, more invasive avenues of reduction.

**Fig. 6:**
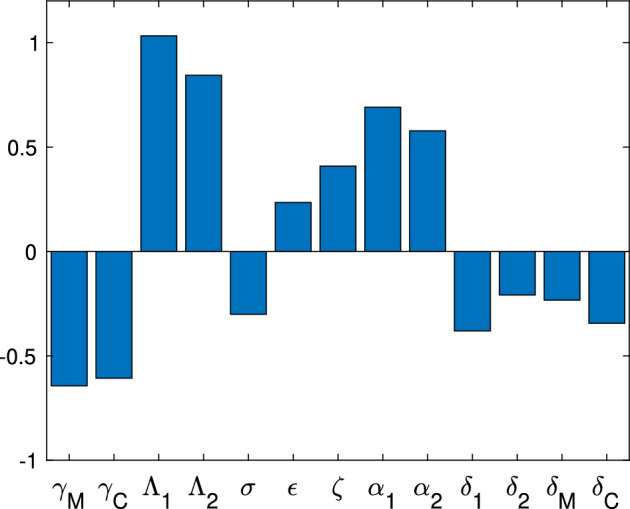
Sensitivity of ${\mathcal {R}}_0$ to parameters. The value of parameters used in computation of sensitivity indices are the same as in Table 2

## Discussion

Due to the complexity of the disease and the vast variety of environmental conditions in which it is found and contracted, the mathematical modeling and dynamical analysis of schistosomiasis plays a crucial role in furthering our understanding of its spread and evaluating potential mitigation measures. In this paper, we formulated a novel, non-autonomous reaction–advection–diffusion model that incorporates hosts (susceptible infected and recovery individuals), snails (healthy and infected snails), miracidia and cercariae, to investigate the impact of spatial heterogeneity and seasonality on the transmission of schistosomiasis. Moreover, as a difference, we add drift terms to describe the effect of advection in water on miracidia and cercariae due to river flow, which has been ignored in previous modelling efforts. Applying the operator semigroup theory and the conception of the next generation operator, we obtain the key threshold found in many epidemic models: the basic reproduction number ${\mathcal {R}}_0$. We then study the asymptotic behavior of ${\mathcal {R}}_0$ for the advection and diffusion rates. Detailed analyses of the global stability of the disease-free periodic solutions and the uniform persistence of the disease are provided by using theory of dissipative and monotonic systems.

To compliment these analytical findings, we also investigate changes in ${\mathcal {R}}_0$ and solution behaviours for some specific cases using numerical simulation. From these simulations, we have observed the following findings and their related biological meaning: (i) We conduct the numerical simulations of the dynamical behavior of system (3.1) in Figs. 2 and 3, which is in lines with the theoretical result mentioned in the corresponding Theorem. (ii) Numerical simulations indicate that seasonality and spatial heterogeneity are two key factors that impact the transmission of schistosomiasis; in Fig. 4, the sensitivity analysis results of ${\mathcal {R}}_0$ for some parameters illustrate that spatial heterogeneity will amplify or dilute schistosomiasis transmission, and it is highly associated with parameters. Moreover, it indicates that seasonality together with the spatial heterogeneity may increase the complexity of schistosomiasis transmission. (iii) We investigate the role of the advection rates $\gamma _M$ and $\gamma _C$ of miracidia and cercariae on the basic reproduction number ${\mathcal {R}}_0$ and dynamical behavior of schistosomiasis outbreak in Figs. 4a, b and 6, respectively. We find that the advection rates $\gamma _M$ and $\gamma _C$ can reduce the value of ${\mathcal {R}}_0$ to some extent and we also notice that the cumulative number of infected individuals decreases with convection rates $\gamma _M$ and $\gamma _C$, which implies that schistosomiasis is generally difficult to break out in fast flowing waters. In other words, the key areas for schistosomiasis prevention and control should be the waters with gentle water flow; alternatively, a cost effective mitigation measure may be to simply encourage bathing/cleaning behaviours in regions with moderate water flow. We were not able to take into account the specific measures such as drug treatment, snail control, cercariae control, improved sanitation and health education, but we will leave such problems for our future study.

This work expands upon previous modelling efforts through inclusion of spatial effect and other environmental factors not previously considered. More precisely, the founding works (Macdonald 1965; Hairston 1965) and subsequent efforts focus primarily on a spatially homogeneous (i.e. through ordinary differential equations) setting. In cases where a partial differential equation setting is used, as in the works (Chan et al. 1995; Zhang et al. 2007), for example, it is most commonly motivated by an age-structure modelling framework. In this sense, the additional “space” dimension included is not physical space as treated in the present work. Motivated by the formulation found in Li et al. (2017), we extend their model to include physical space in addition to the influence of seasonality. In our case, space is taken to be a one-dimensional, bounded domain (0, *H*), with boundary conditions chosen such that the model corresponds most closely with schistosomiasis spread in a river system. Naturally, diffusion is a key factor, which includes cases of spatially dependent diffusion processes. Through its construction, we are also able to include advective forces caused by flowing water in the river. Inclusion of such environmental effects are important to further our understanding of disease spread in areas such as villages along the Yangze River region. One interesting finding is the effect of slow or fast flowing water on disease spread; our findings are consistent with case studies, where schistosomiasis is less prevalent in areas with faster flowing water when compared to areas with slower water flow. Such findings can provide further insights into less intrusive mitigation measures. For example, chemicals used to eliminate the snail population from sources of fresh water may have a negative impact on other local species. Furthermore, without continual treatment of the water source, it is likely that the snail population will inevitably return (Centers for Disease Control and Prevention 2020).

Despite the novel extensions presented here, there are numerous areas ripe for further study. As performed in the works of Chan et al. (1995), Zhang et al. (2007), it would be interesting to consider an age-structure in addition to spatial effects considered here. Our work also does not include many aspects that we know to be relevant to disease transmission: social, psychological, and economic factors should not be entirely ignored. It would be a challenging but potentially fruitful task to incorporate these additional effects to determine optimal control strategies as they relate to explicit spatial effects. More broadly, the modelling approach presented here could be applied to a wider range of scenarios which include the transmission of disease via a river system.
